# 2D Programmable Photodetectors Based on WSe_2_/h‐BN/Graphene Heterojunctions

**DOI:** 10.1002/advs.202417300

**Published:** 2025-04-04

**Authors:** Zhihao Wang, Jialing Jian, Zhengjin Weng, Qianqian Wu, Jian Li, Xingyu Zhou, Wei Kong, Xiang Xu, Liangliang Lin, Xiaofeng Gu, Peng Xiao, Haiyan Nan, Shaoqing Xiao

**Affiliations:** ^1^ Engineering Research Center of IoT Technology Applications (Ministry of Education) School of Integrated Circuits Jiangnan University Wuxi 214122 China; ^2^ School of Engineering Westlake University Hangzhou 310030 China; ^3^ School of Chemical and Material Engineering Jiangnan University Wuxi 214122 China; ^4^ Laboratoire Ondes et Matière d'Aquitaine (LOMA)‐UMR 5798 CNRS Talence F‐33400 France

**Keywords:** 2D photodetector, fast response, non‐volatile, programmable, semi‐floating gate

## Abstract

Programmable photovoltaic photodetectors based on 2D materials can modulate optical and electronic signals in parallel, making them particularly well‐suited for optoelectronic hybrid dual‐channel communication. This work presents a programmable non‐volatile bipolar semi‐floating gate photovoltaic photodetector (SFG‐PD) constructed using tungsten diselenide (WSe_2_), hexagonal boron nitride (h‐BN), and graphene (Gra). By controlling the voltage pulses applied to the control gate, the device generates opposing built‐in electric field junctions (p^+^‐p and n‐p junctions), enabling reversible switching between positive and negative light responses with a rapid response time of up to 2.02 µs. Moreover, the application of this device is demonstrated in dual‐channel optoelectronic hybrid communication, offering a practical solution for achieving high‐speed, large‐capacity, low‐loss, and secure multi‐channel communication.

## Graphical abstract

A programmable non‐volatile bipolar semi‐floating gate photovoltaic photodetector is developed, enabling reversible light response switching and improved response speed. This capability facilitates the successful implementation of the device in dual‐channel optoelectronic hybrid communication, fulfilling the performance demands of high‐speed, large‐capacity, low‐loss, and secure operation.

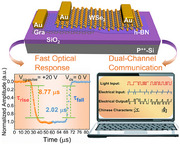

## 1 Introduction

Traditional electronic communication systems face limitations such as bandwidth bottlenecks and low energy efficiency, making it challenging to meet the demands for data transmission speed, capacity, and latency brought about by the rapid development of artificial intelligence (AI) and big data technologies.^[^ ^1^ ^]^ Optoelectronic hybrid dual‐channel communication offers a potential solution by leveraging the advantages of optical and electronic signals to achieve high‐speed, high‐capacity, and low‐power data transmission. Programmable photovoltaic photodetectors based on atomically thin 2D materials^[^ ^2^ ^]^ can modulate optical and electronic signals in parallel, making them particularly well‐suited for such systems.^[^ ^3^ ^]^

Programmable photovoltaic photodetectors based on 2D materials can establish built‐in electric field junctions through electrostatic doping, enabling dynamic modulation of the majority of internal carriers. This approach overcomes the limitations of traditional photovoltaic photodetectors, such as unidirectional built‐in electric fields, low light absorption, and fixed carrier transport behaviors.^[^ ^4^ ^]^ It also enables the formation of various types of junctions, including p^+^‐p,^[^ ^5^ ^]^ n‐p,^[^ ^5^ ^]^ n^+^‐n,^[^ ^6^ ^]^ and Schottky junctions,^[^ ^7^ ^]^ paving the way for the practical use of programmable photovoltaic photodetectors in optoelectronic hybrid communication systems.^[^ ^8^ ^]^

Programmable photovoltaic photodetectors typically rely on electrostatic doping, achieved through continuous or pulsed gating. This approach allows precise control over the direction of the built‐in electric field, enabling seamless switching between positive and negative responses. Examples include homojunction‐based volatile photovoltaic detectors, such as those made from WSe_2_,^[^ ^9^ ^]^ BP,^[^ ^10^ ^]^ MoTe_2_,^[^ ^11^ ^]^ and non‐volatile devices like MoTe_2_.^[^ ^6^ ^]^ Compared to volatile photovoltaic photodetectors controlled by continuous gating, non‐volatile devices utilizing split gating consume less power.^[^ ^6^ ^]^ Among these, non‐volatile photovoltaic photodetectors based on a semi‐floating gate (SFG) structure stand out due to their well‐defined dimensions, extended retention time, rapid response, and cost‐effectiveness.^[^ ^5^, ^12^ ^]^

To further enhance the response speed, we developed a programmable non‐volatile bipolar semi‐floating gate photovoltaic photodetector (SFG‐PD) using WSe_2_, hexagonal boron nitride (h‐BN), and graphene (Gra). The WSe_2_ SFG‐PD device demonstrates exceptional performance and unique functionality, achieving rapid positive and negative self‐driven light (637 nm) responses of 8.77 µs / 2.02 µs and 5.92 µs / 7.80 µs, respectively, under +20 V and −20 V gate voltage pulses. Additionally, the self‐driven device exhibits high responsivities of 2.76 A/W and 1.63 A/W, high specific detectivity of 7.86 × 10^11^ Jones and 4.42 × 10^11^ Jones, low noise equivalent power of 4.76 × 10^−15^ W/Hz^1/2^ and 8.47 × 10^−15^ W/Hz^1/2^, under these two gate voltage pulses, respectively, and excellent retention performance exceeding 2 × 10^3^ s. Furthermore, we utilized the WSe_2_ SFG‐PD device to convert binary optoelectronic mixed signals into balanced ternary electrical signals in real‐time, enabling high‐speed, large‐capacity, low‐loss, and secure dual‐channel optoelectronic hybrid communication. This innovative approach positions the WSe_2_ SFG‐PD as a promising solution for multi‐channel communication. Its advanced optoelectronic hybrid communication capabilities provide a practical and efficient means to address the demands for high‐speed, high‐capacity, and low‐power data transmission in the era of AI and big data, paving the way for future advancements in communication technologies.

## 2 Results and Discussion

### 2.1 Device Structure and Microscopic Characterization

**Figure 1:**
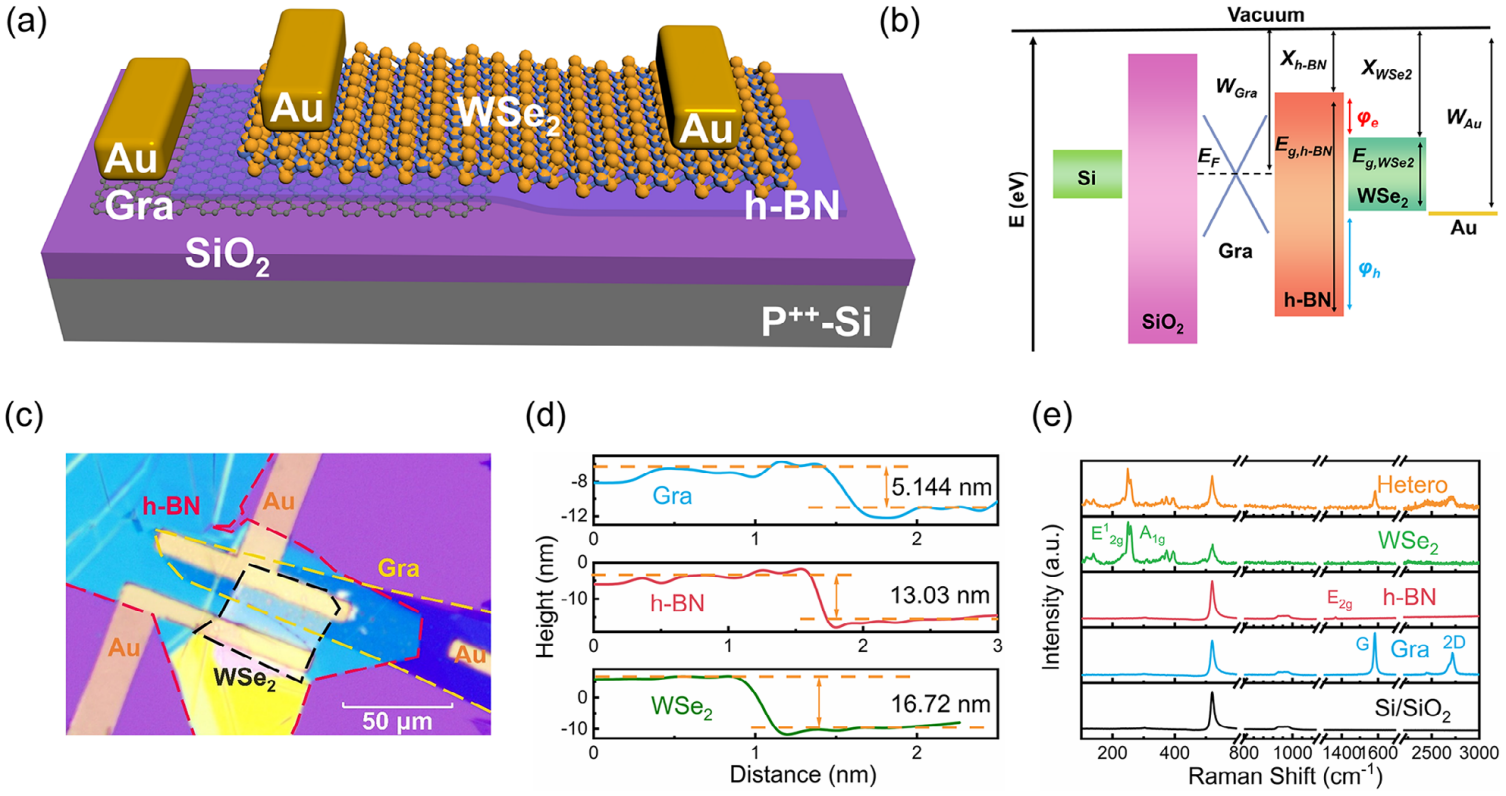
Conceptual illustration and characterization of the WSe_2_/h‐BN/Gra heterojunction. a) Schematic diagram of the WSe_2_/h‐BN/Gra semi‐floating gate photodetector (WSe_2_ SFG‐PD). b) Flat energy bands of the Au/WSe_2_/h‐BN/graphene heterostructure. c) Optical microscope image of the fabricated WSe_2_ SFG‐PD device. d) Extracted heights of the Gra, h‐BN, and WSe_2_ flakes corresponding to the blue, orange, and green lines of proposed devices (in Figure S2, Supporting Information). e) Raman spectrum of the WSe_2_/h‐BN/Gra heterostructure photodetector.

A schematic diagram of the non‐volatile SFG‐PD based on the WSe_2_/h‐BN/Gra heterostructure is depicted in **Figure** **1**a, where WSe_2_, h‐BN, and Gra flakes serve as the channel, tunneling layer, and SFG, respectively. The corresponding flat band diagram of the WSe_2_/h‐BN/Gra heterostructure is shown in Figure 1b. Here, W_Au_ = 5.1 eV,^[^ ^13^ ^]^ W_Gra_ = 4.6 eV,^[^ ^13^ ^]^ and W_WSe2_ = 4.89 eV are the work functions of Au, graphene, and WSe_2_, respectively (as shown in Figure S1, Supporting Information). Additionally, X_WSe2_ = 3.5–4.0 eV and X_h‐BN_ = 2−2.3 eV^[^ ^13^ ^]^ are the electron affinities of WSe_2_ and h‐BN, respectively, while the bandgaps of WSe_2_ (E_g,WSe2_) and h‐BN (E_g,h‐BN_) are 1.4 and 5.96 eV,^[^ ^5^ ^]^ respectively. The working principle of this structure primarily relies on the band alignment of the heterojunction and the quantum tunneling effect WSe_2_, as the channel material, can effectively absorb photons and generate charge carriers, while h‐BN, serving as the tunneling layer, allows for the rapid tunneling of charge carriers into the SFG (Gra) under appropriate gate voltage pulses, thereby generating image charge in the channel material and creating a tunable built‐in electric field. This design allows for efficient generation and detection of current under illumination, while the non‐volatile characteristics enable the photodetector to retain the previous signal state even after being turned off. The fabrication process of the device is described in detail in the experimental section, as shown in Figure S2 (Supporting Information). Figure 1c shows the optical microscope image of a typical non‐volatile SFG‐PD based on the WSe_2_/h‐BN/Gra heterostructure, where WSe_2_, h‐BN, and Gra flakes are indicated by black, red, and yellow dashed lines, respectively. The thickness of WSe_2_, h‐BN, and Gra was characterized using atomic force microscopy (AFM), with the flake thicknesses measured at 16.72, 13.03, and 5.14 nm, respectively, as shown in Figure 1d (the device image under AFM is shown in Figure S3, Supporting Information). First, single‐layer graphene limits its ability to store a significant number of carriers due to its low density of states,^[^ ^5^ ^]^ while thicker graphene can cause the h‐BN above the heterojunction to fracture. Therefore, the selection of graphene thickness is crucial. After extensive experiments, a multilayer graphene thickness of 5.14 nm was chosen as the floating gate.^[^ ^14^ ^]^ Additionally, h‐BN, which has a wide bandgap (≈6 eV), is commonly used as a dielectric tunneling layer. For 2D material floating gate devices, the tunneling layer thickness is typically set between 10 and 20 nm.^[^ ^15^ ^]^ Considering this, the thickness of the h‐BN flake was chosen to be ≈13.03 nm to achieve ultrafast operation speed and excellent retention performance. Figure 1e displays the Raman spectrum of the WSe_2_/h‐BN/Gra heterostructure. The WSe_2_ flakes exhibit two distinct peaks at 250 and 258 cm^−1^, corresponding to the in‐plane (E^1^_2_ _g_) and out‐of‐plane (A_1_ _g_) vibrational modes, respectively. The characteristic peak of h‐BN is located at 1366 cm^−1^, corresponding to the in‐plane (E_2_ _g_) vibrational mode. Furthermore, the peaks at 1582 cm^−1^ (G) and 2716 cm^−1^ (2D) are two typical characteristic peaks of multi‐layer graphene. This phenomenon shows that no additional discernible Raman peaks were observed, affirming the absence of defects introduced during the fabrication process. In addition, to further assess the structural properties and quality of the WSe_2_, h‐BN, and Gra materials, X‐ray diffraction (XRD) measurements were conducted. The results presented in Figure S4 (Supporting Information) reveal that these materials display an ordered layered structure with well‐aligned interlayer stacking, minimal defects, and high crystallinity, further confirming their superior quality characteristics.

### 2.2 Device Performance and Mechanism

**Figure 2:**
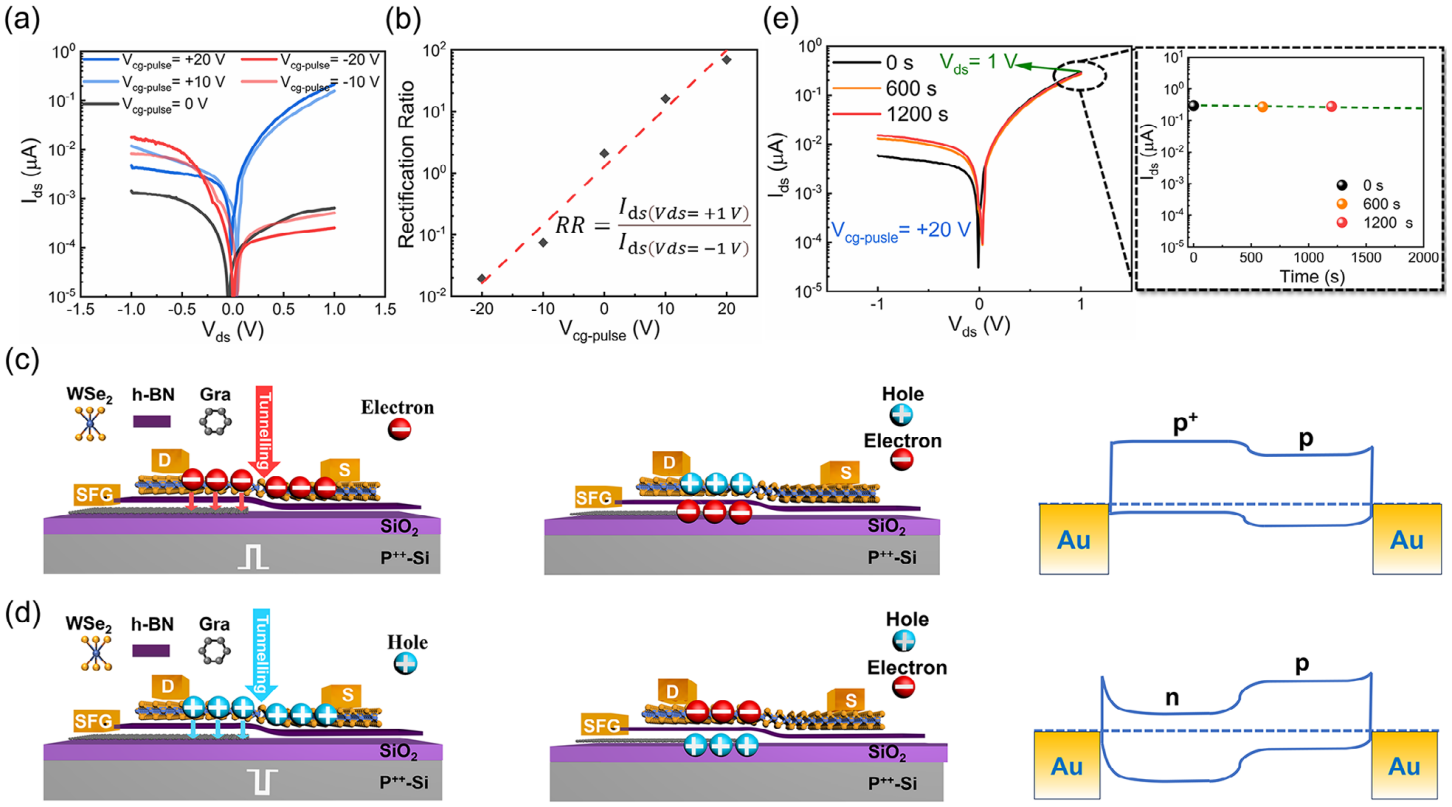
Implementation of non‐volatile p^+^‐p/n‐p junctions in the WSe_2_/h‐BN/Gra heterojunction. a) Semi‐logarithmic I_DS_‐V_DS_ curves of WSe_2_ under different gate voltage pulses applied to Si. b) Extraction and fitting of the rectification ratio of WSe_2_ corresponding to different gate voltage pulses based on the proposed device. c) Operation diagram of the WSe_2_ SFG‐PD device under positive V_cg_ applied and released to the Si control gate (left), tunneling charges in the SFG layer inducing mirror charge formation in the channel material (middle), and the energy band diagram of the p^+^‐p homojunction device (right). d) Operation diagram of the WSe_2_ SFG‐PD device under negative V_cg_ applied and released to the Si control gate (left), tunneling charges in the SFG layer inducing mirror charge formation in the channel material (middle), and the energy band diagram of the n‐p homojunction device (right). e) Time‐dependent semi‐logarithmic I_DS_–V_DS_ curve of WSe_2_ under a +20 V gate voltage pulse applied on Si. Inset: Fitting curve of I_DS_ as a function of time for WSe_2_ under a +20 V gate voltage pulse with V_DS_ = +1 V applied to Si.

The electrical characteristics were evaluated using a Si substrate as the control gate (V_cg_) for channel programming and erasing. In this study, the pulse duration was uniformly set to 1 s to ensure saturation at a specific V_cg_ pulse level, with Gra serving as the storage charge in the SFG. As shown in the output curve of **Figure** **2**a, a pulsed gate voltage of V_cg‐pulse_ = ±20 V (Per increment |10| V) was applied to the Si substrate, resulting in opposite rectifying characteristics in the WSe_2_ SFG‐PD device under the ±V_cg‐pulse_. The fitting curves of the rectification ratio under different V_cg‐pulse_ conditions show that the rectification ratio exhibits an almost linear dependence on the gate V_cg‐pulse_, specifically being ≈10^−2^ at V_cg‐pulse_ = −20 V and ≈10^2^ at V_cg‐pulse_ = +20 V in Figure 2b. This indicates that the modulation levels and mechanisms for different ±V_cg‐pulse_ may be similar, the effect observed in the WSe_2_ SFG‐PD device is attributed to charge injection/capture in the SFG and the channel heterojunction configurations (p^+^‐p and n‐p).

As shown in Figure 2c, during the application of V_cg_ > 0 V, electrons rapidly accumulate in WSe_2_ and tunnel through the h‐BN layer to reach the SFG, where they are stored (left image). After transitioning from V_cg_ > 0 V to V_cg_ = 0 V (where a complete positive gate pulse has been formed), the accumulated electrons in WSe_2_ disappear, and the stored tunneling electrons in the SFG induce image charge holes in the WSe_2_ above (middle image). Consequently, holes accumulate in the portion of WSe_2_ above the SFG, lowering the Fermi level and forming a p^+^‐p junction, as depicted in the band structure in the right panel of Figure 2c. Similarly, as shown in Figure 2d, during the application of V_cg_ < 0 V, holes rapidly accumulate in WSe_2_ and tunnel through the h‐BN layer to reach the SFG, where they are stored (left image). After transitioning from V_cg_ < 0 V to V_cg_ = 0 V (where a complete negative gate pulse has been formed), the accumulated holes in WSe_2_ disappear, and the stored tunneling holes in the SFG induce image charge electrons in the WSe_2_ above (middle image). Therefore, electrons accumulate in the portion of WSe_2_ above the SFG, raising the Fermi level and forming an n‐p junction, as depicted in the band structure in the right panel of Figure 2d. If Gra is grounded, the rectifying characteristics of WSe_2_ will disappear. Notably, charge modulation within the h‐BN/Gra van der Waals (vdWs) floating gate can be achieved at a speed of 20 ns through the improved Fowler‐Nordheim tunneling effect.^[^ ^16^ ^]^ To characterize the device stability, as shown in Figure 2e, the output characteristics of the device were tested at 0, 600, and 1200 s after applying V_cg‐pulse_ = +20 V. It is evident that I_DS_ (V_DS_ = −1 V) gradually increases over time, attributed to a small number of electrons in the portion of WSe_2_ above the SFG tunneling through h‐BN and being captured by defects in SiO_2_, releasing slowly.^[^ ^17^ ^]^ Similarly, after applying V_cg‐pulse_ = −20 V, a small number of holes in the portion of WSe_2_ above the SFG tunnel through h‐BN when the negative gate pulse is applied and are captured by defects in SiO_2_, releasing slowly.^[^ ^17^ ^]^ As a result, I_DS_ (V_DS_ = −1 V) gradually decreases over time (as shown in Figure S5, Supporting Information). The changes in I_DS_ (V_DS_ = 1 V) over time for V_cg‐pulse_ = ±20 V are fitted in Figure 2e and Figure S5 (Supporting Information), clearly indicating that the carriers stored in the SFG can be retained for over 2000 s. To further evaluate the stability of the device, we tested the performance of the WSe_2_ SFG‐PD device over 1000 cycles of periodic gate voltage pulses. As shown in Figure S6a (Supporting Information), the device maintains stable and reversible light response characteristics under these periodic gate voltage pulses, indicating that it does not exhibit significant performance degradation during repeated operation. This behavior demonstrates that the WSe_2_ SFG‐PD device possesses excellent operational stability, capable of withstanding long‐term periodic operation without substantial performance decay. Furthermore, Figure S6b (Supporting Information) presents the error bar curve extracted from Figure S6a (Supporting Information), where it is clear that, after 1000 gate voltage pulses, the performance fluctuations of the device remain within a narrow range, further confirming the high stability and reliability of the device. Based on these test results, we conclude that the device demonstrates high durability and long‐term stability, making it well‐suited to withstand frequent operations and environmental changes in practical applications.

To assess the quality of the device, it is necessary to evaluate the number of recombination centers and trap states through the carrier transport process in the homojunction. The transport in the homojunction can be fitted using the Shockley diode equation

(1) $$I_{D}=\frac{nV_{T}}{R_{s}}\;W\left[\frac{I_{0}R_{s}}{nV_{T}}\exp\left(\frac{V_{DS}+I_{0}R_{s}}{nV_{T}}\right)\right]-I_{0}$$

here, *I_0_* is the reverse saturation current, *V_T_* = k_B_ *T*/q is the thermal voltage at temperature *T*, R_s_ is series resistance, where k_B_ is the Boltzmann constant, q is the electronic charge, *W* is the Lambert *W* function, and *n* is the ideality factor.^[^ ^5^ ^]^ The ideality factor *n* describes the diffusion and recombination behavior of charge carriers as they traverse the depletion region. It is commonly used to characterize the imperfections in real transistor junctions, as recombination centers are invariably present in these regions. It typically ranges from 1 to 2 depending on the fabrication process, interfaces, and semiconductor materials. For an “ideal” diode, there are no recombination centers in the junction depletion region, thus the ideality factor *n* equals 1. From the I_DS_‐V_DS_ curve (see Figure S7, Supporting Information), we calculated *n* = 1.13 for the p^+^‐p junction and *n* = 1.24 for the n‐p junction. Here, we conclude that the charge carriers in the p⁺‐p junction at V_cg‐pulse_ = +20 V and the n‐p junction at V_cg‐pulse_ = −20 V are predominantly driven by diffusion, with a mixture of diffusion and recombination processes during transport. Since the ideality factor *n* in the p^+^‐p junction is closer to 1 than that in the n‐p junction, this indicates that there are fewer recombination centers and trap states in the p^+^‐p junction.

Previous studies^[^ ^18^ ^]^ have shown that at lower gate voltages, direct tunneling dominates, while at higher gate voltages, the Fowler–Nordheim (F–N) mechanism governs the tunneling process. The current equation for F–N tunneling can be expressed as follows:^[^ ^18^ ^]^

(2) $$I\;\left(V\right)=\frac{A_{eff}q^{3}mV^{2}}{8\pi h\phi_{B}d^{2}m^{*}}\;\exp\left(\frac{-8\pi \sqrt{2m^{*}}\phi_{B}^{\frac{3}{2}}d}{3\mathrm{hq}V}\right)$$

where *V*, *A_eff_*, *ϕ_B_*, q, *m*, m*, *d*, and h represent the applied voltage, effective contact area, barrier height, electron charge, mass of a free electron, mass of tunneling electrons in h‐BN (m*/*m* = 0.26),^[^ ^19^ ^]^ thickness of the tunneling layer, and Planck's constant, respectively. The equation can be rewritten as:

(3) $$\ln\left(\frac{I\left(V\right)}{V^{2}}\right)=\ln A-B\cdot \frac{1}{V}$$

where

(4) $$A=\frac{A_{eff}q^{3}m}{8\pi h\phi_{B}d^{2}m^{*}},B\;=\frac{8\pi \sqrt{2m^{*}}\phi_{B}^{\frac{3}{2}}d}{3\mathrm{hq}}$$

The F–N tunneling current was tested by applying voltage to the drain and SFG electrodes to illustrate the carrier tunneling mechanism. Figure S8a (Supporting Information) shows the test results, which indicate that direct tunneling dominates when V_DS_ ≤ 5.1 V, while F–N tunneling prevails when V_DS_ > 5.1 V. Additionally, the inset shows a schematic structure of the tested device. Figure S8b (Supporting Information) displays the F−N plot of the I−V curves obtained from the tests, where the linear relationship indicates that the F−N tunneling mechanism dominates in our device.^[^ ^20^ ^]^

### 2.3 Reconfigurable Photovoltaic Characteristics

**Figure 3:**
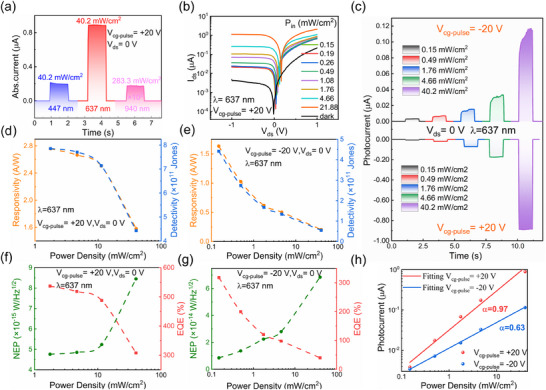
Reconfigurable photovoltaic characteristics in the WSe_2_ SFG‐PD device. a) Measurement of the switchable electro‐optical response of the WSe_2_ SFG‐PD device at different operating wavelengths under varying incident light powers and at 0 V bias. b) Semi‐logarithmic output curves under different input optical powers (P_in_) with a +20 V voltage pulse applied to Si substrate at 637 nm and 0 V bias. c) Optical response of the WSe_2_ SFG‐PD structure under a ±20 V voltage pulse on Si substrate at 637 nm illumination, 0 V bias, with power switching from 0.038 to 10.23 µW. d,e) Photodetector performance of the device at V_cg‐pulse_ = ±20 V, 0 V bias, and 637 nm, corresponding to responsivity (R) and detectivity (D*). f,g) Photodetector performance of the device at V_cg‐pulse_ = ±20 V, 0 V bias, and 637 nm, corresponding to noise equivalent power (NEP) and external quantum efficiency (EQE). h) Fitting relationship between photocurrent and optical intensity under V_cg‐pulse_ = ±20 and 0 V bias at 637 nm.

Further exploration was conducted on the photovoltaic properties of the WSe_2_ SFG‐PD with reconfigurable light response. **Figure** **3**a shows the measured photocurrent at V_cg‐pulse_ = +20 V, 0 V bias, and at different wavelengths. Under illumination of visible light (447 and 637 nm) and near‐infrared light (940 nm), the device exhibits a significantly higher photocurrent at 637 nm compared to other wavelengths, even at lower input power (Figure S9, Supporting Information). Therefore, the following experiments were all conducted under 637 nm light illumination. The band alignment in the WSe_2_ channel facilitates the formation of p^+^‐p or n‐p homojunctions under ±V_cg‐pulse_ tuning, resulting in significant photovoltaic effects with reversed built‐in electric fields. Therefore, under 637 nm laser illumination, both reconstructive rectifying behavior and reversible positive/negative photovoltaic behavior can be observed, as shown in Figure 3b and Figure S10 (Supporting Information). Figure 3c shows the dynamic light response at 0 V bias with V_cg‐pulse_ = ±20 V, demonstrating fast self‐driven photocurrents with opposite signals for the two modes (p^+^‐p and n‐p junctions), reaching photocurrents (I_ph_) of up to 0.89 and 0.12 µA under 10.23 µW laser illumination, and switch ratios (I_on_/I_off_) of 1600 and 200. This indicates that the current change in the switch state of the detector is significant, usually reflecting good sensitivity and responsiveness. Moreover, Figure 3d,e shows the responsivity of the proposed device is ≈2.76 and 1.63 A/W under V_cg‐pulse_ = ±20 and 0 V bias, while the detectivity is 7.86 × 10^11^ and 4.42 × 10^11^ Jones, respectively. The device's higher responsivity exceeds that of similar SFG‐PD devices.^[^ ^6^ ^]^ When V_cg‐pulse_ = +20 and at 1 V bias, the device exhibits maximum responsivity and detectivity of 292.20 A/W and 4.16 × 10^12^ Jones (as shown in Figure S11a, Supporting Information), indicating its excellent ability to detect weak light signals. To further test the signal‐to‐noise ratio, we measured the noise equivalent power (NEP) of the device. Figure 3f,g displays the NEP (left axis) and external quantum efficiency (right axis) under V_cg‐pulse_ = ±20 and 0 V bias. The noise equivalent powers are 4.76 × 10^−15^ W/Hz^1/2^ and 8.47 × 10^−15^ W/Hz^1/2^, while the external quantum efficiencies are 537% and 318%, respectively. When V_cg‐pulse_ = +20 and at 1 V bias, the device achieves minimum noise equivalent power and maximum external quantum efficiency of 8.99 × 10^−16^ W/Hz^1/2^ and 56 900% (as shown in Figure S11b, Supporting Information), demonstrating its exceptional capability to convert light signals into electrical signals.

We can derive the relationship between photocurrent and light intensity, as shown in Figure 3h. We fitted the data using a power‐law equation of the form I_ph_ = cP^α^. For the device, the *α* value lies between 0 and 1; the closer *α* is to 1, the fewer trap states are present in the material, resulting in less loss of carriers due to recombination, with the photoconductive effect dominating the photocurrent. Conversely, the closer *α* is to 0, the more trap states are present, causing the photocurrent to be determined by the photogating effect.^[^ ^21^ ^]^ When the *α* values for the two different homojunctions (p^+^‐p and n‐p) generated at V_cg‐pulse_ = ±20 V were 0.97 and 0.63, respectively, it indicates that the device's photocurrent is dominated by the photoconductive effect in both modes. The *α* value for the p^+^‐p junction mode is greater than that for the n‐p junction mode, indicating that there are fewer trap states in the p^+^‐p junction mode, leading to less carrier loss during recombination. This is consistent with our conclusions drawn from the ideality factors in both modes.

**Figure 4:**
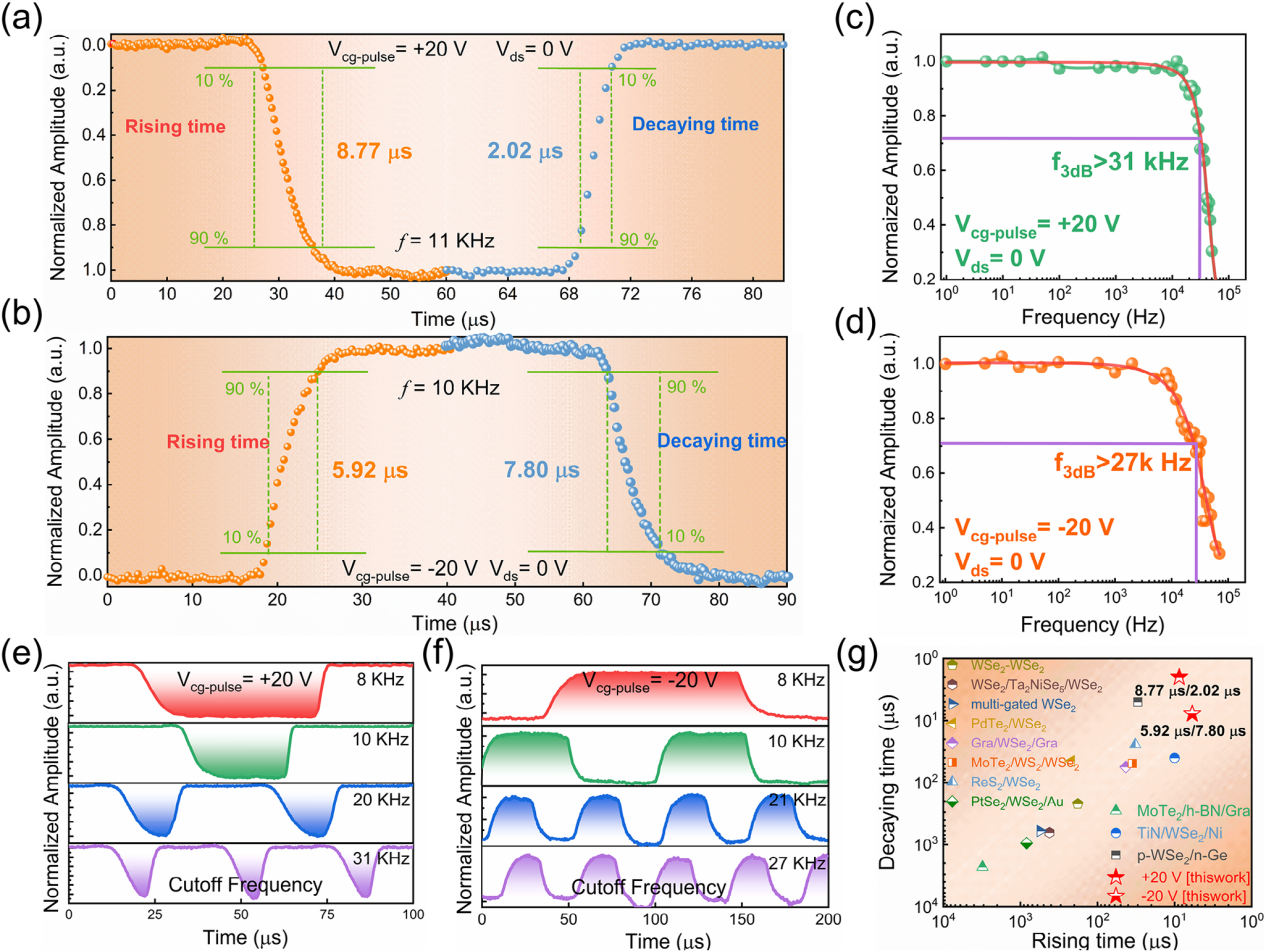
Electro‐optical response of the WSe_2_ SFG‐PD device under 637 nm illumination, with V_cg‐pulse_ = ±20 and 0 V bias. a) Rise time and fall time of the WSe_2_ SFG‐PD under V_cg‐pulse_ = +20 and 0 V bias. b) Rise time and fall time of the WSe_2_ SFG‐PD under V_cg‐pulse_ = −20 and 0 V bias. Variation of normalized photocurrent in the WSe_2_ SFG‐PD device with laser modulation frequency under c) V_cg‐pulse_ = +20 and 0 V bias. d) V_cg‐pulse_ = −20 and 0 V bias. Normalized optical response characteristics of the WSe_2_ SFG‐PD to different frequency pulse signals under e) V_cg‐pulse_ = +20 and 0 V bias. f) V_cg‐pulse_ = −20 and 0 V bias. g) Comparison of response times of different types of WSe_2_ heterostructure photodetectors.

The response bandwidth is a key performance indicator directly related to the response speed of the photodetector. The response speed is a key advantage of the WSe_2_ SFG‐PD device, reflecting its ability to track rapidly switching optical signals. Here, we use a 637 nm laser irradiation setup to characterize the time‐ dependent optical response behavior. The rise time (τ_r_) and decay time (τ_d_) are defined as the time taken for the photocurrent to rise from 10% to 90% of its peak value or to decay from 90% to 10%. **Figure** **4**a shows the optical response times τ_r_ = 8.77 µs and τ_d_ = 2.02 µs at V_cg‐pulse_ = +20 V with a 0 V bias. Figure 4b displays the optical response times τ_r_ = 5.92 µs and τ_d_ = 7.80 µs at V_cg‐pulse_ = −20 V with a 0 V bias. It is evident that the response speeds under the built‐in electric fields of the two different modes (p^+^‐p and n‐p junctions) are very fast and quite similar. This can be attributed to the introduction of a sacrificial Se layer between Au and WSe_2_ to form vdWs contacts (as shown in Figure S12, Supporting Information), avoiding defects at the electrode interface made by conventional metal deposition methods, and the limitation of Fermi level pinning. The reduction of defects at the electrode interface decreases the impact of lattice scattering at the metal‐semiconductor contact, thereby enhancing the speed of carrier collection. Additionally, the vdWs contact formed between WSe_2_ and h‐BN also reduces lattice scattering at the interface, accelerating the migration speed of photogenerated carriers. On the other hand, the wide depletion region in WSe_2_ and the enhanced built‐in electric field can accelerate the rapid separation of photogenerated carriers. Furthermore, the essence of the built‐in electric field in this device is the generation of image charges in the WSe_2_ above due to the stored tunneling charges in the SFG. This electrostatic doping does not introduce defect states into the channel material during doping, as with methods like ion implantation, thus reducing losses and scattering effects due to defect states within WSe_2_. Furthermore, we investigate the frequency response of the photodetector by modulating the frequency of the incident light signal. The normalized response of the photodetector to pulsed 637 nm laser over a wide frequency range of 1 Hz–100 kHz is shown in Figure 4c,d. The cutoff frequency is defined as the frequency at which the output signal decreases to −3 dB of the initial output signal as the pulse laser frequency increases. In the case of our WSe_2_ SFG‐PD device, the cutoff frequency was found to be >31 kHz at V_cg‐pulse_ = +20 V and >27 kHz at V_cg‐pulse_ = −20 V. Figure 4e,f shows the rapid and consistent switching of photocurrent in the WSe_2_ SFG‐PD device under different switching frequencies at V_cg‐pulse_ = ±20 V. This reveals the capability of the photodetector to detect ultrafast optical signals and operate at high frequencies with good stability and reproducibility. However, the device's response degrades at higher frequencies. Figure 4g illustrates the comparison of the response speed performance of the WSe_2_ SFG‐PD device developed in this study with that of other previously reported WSe_2_ composite‐structure photodetectors. The device achieves a maximum response speed of 2.02 µs under 637 nm illumination, surpassing the response speed of existing composite‐structure WSe_2_ photodetectors,^[^ ^6^, ^22^, ^23^ ^]^ further highlighting the significant advantages of the WSe_2_ SFG‐PD device in practical applications.

At the same time, we investigated the effect of different thicknesses of WSe_2_ on the performance of this structural device and observed some interesting phenomena. Here, we fabricated WSe_2_ SFG‐PD devices with three thickness categories: thin (below 10 nm), medium (10–30 nm), and thick (over 30 nm), and tested the semi‐logarithmic I_DS_‐V_DS_ curves of these devices under different gate pulses. The rectifying behavior of WSe_2_ devices varies significantly with thickness and applied voltage. For thin WSe_2_ devices (Figure S13a, Supporting Information), a positive pulse (V_cg‐pulse_ >0 V) leads to an intensified rectifying effect, achieving a ratio near 10^3^, while a negative pulse (V_cg‐pulse_ <0 V) results in a diminishing rectifying effect, exhibiting low‐pass behavior. In medium‐thickness WSe_2_ devices (Figure S14a, Supporting Information), a positive pulse reveals a clear forward rectification effect with a ratio approaching 10^2^, whereas a negative pulse displays a strong reverse rectification effect with a ratio ≈10^−2^, enabling both positive and negative light responses suitable for dual‐channel communication. Thick WSe_2_ devices (Figure S15a, Supporting Information) show a progressive reduction in rectifying effect under positive pulses, leading to high‐pass behavior, but maintain a robust rectifying effect with a negative pulse, achieving a ratio close to 10^−3^. Based on the experimental observations, we conclude that the Fermi level of WSe_2_ shifts downward as the thickness of the flakes increases, with thinner WSe_2_ exhibiting n‐type characteristics.^[^ ^24^ ^]^ The channel region of thinner WSe_2_ experiences a stronger influence from charged interface impurities, which suppresses hole conduction and results in n‐type behavior. As the thickness increases, these charged impurities become screened, allowing WSe_2_ to display intrinsic properties. Additionally, the density of states increases, leading to a reduction in the Schottky barrier. This shift allows the Fermi level of WSe_2_ to align more closely with that of the metal. Therefore, we select WSe_2_ flakes with a thickness of 10–30 nm to serve as the channel for dual‐channel communication devices.

### 2.4 Dual‐Channel Photoelectric Hybrid Communication

**Figure 5:**
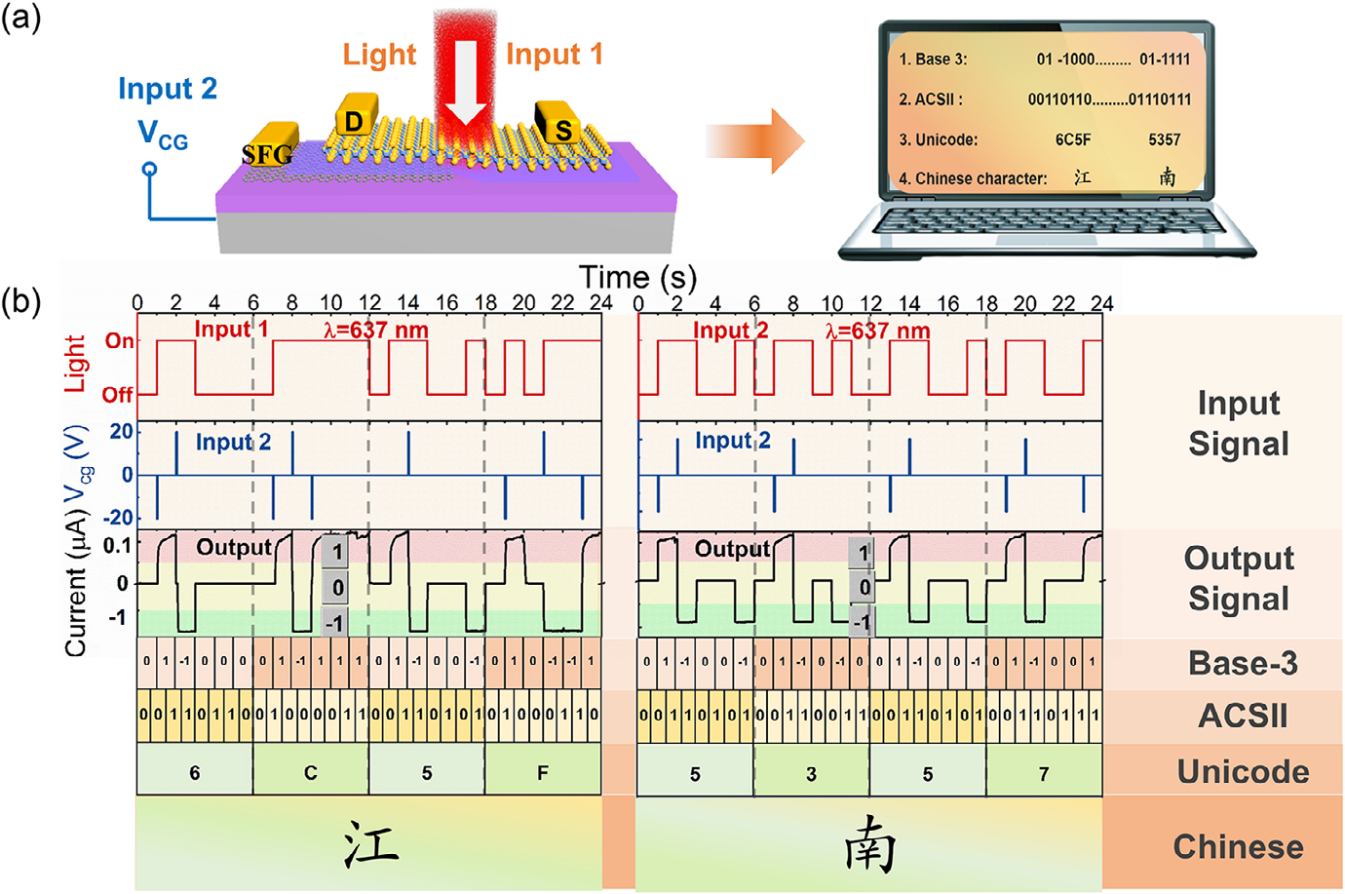
Dual‐channel optoelectronic hybrid communication. a) Schematic illustration of dual‐channel optoelectronic hybrid communication based on the WSe_2_ SFG‐PD device. b) This dual‐channel optoelectronic hybrid communication system transmits input and output signals containing information streams “6C5F” and "5357″ and decodes Chinese characters through Unicode encoding.

Recognizing the importance of programmable photovoltaic photodetectors in communication, we applied the WSe_2_ SFG‐PD device in optoelectronic hybrid communication using a dual‐channel parallel time‐interleaved mode. Using the unique functionalities of the device's special structure, we have realized the real‐time output of binary optoelectronic mixed input signals as balanced ternary electrical signals. The analog electrical signals output by the device are then converted into digital electrical signals through computer programming, which are subsequently transformed into ASCII and Unicode, ultimately resulting in Chinese characters, as shown in **Figure** **5**a. Using the WSe_2_ SFG‐PD as a receiver for optical and electrical signals, we synchronized the collection of the 637 nm laser's optical pulse signal and a voltage pulse signal with an amplitude of ±20 V lasting 5 ms. This resulted in the output of a balanced ternary electrical signal data stream through the output photocurrent (Regulation: A positive direction of the output photocurrent greater than |10| nA is considered logic ‘1′, a value less than |10| nA is considered logic ‘0′, and a negative direction of the output photocurrent signal greater than |10| nA is considered logic ‘‐1′). For example, when the WSe_2_ SFG‐PD device receives a continuous voltage pulse signal of −20 V for 5 ms, it forms an n‐p junction. At the same time, when the optical pulse signal illuminates the junction region of the device, the built‐in electric field causes the device to output a photocurrent that is positive and greater than |10| nA, which is considered as logic “1”. However, when the WSe_2_ SFG‐PD device receives a continuous voltage pulse signal of +20 V for 5 ms, it forms a p^+^‐p junction. At the same time, when the optical pulse signal illuminates the junction region of the device, the built‐in electric field of the p^+^‐p junction, which is oriented opposite to that of the previous n‐p junction, causes the device to output a photocurrent that is negative and greater than |10| nA, which is considered as logic “‐1”. In the absence of illumination, the device outputs a current less than |10| nA, which is regarded as logic “0”. Therefore, by inputting specific optical pulse signals and electrical pulse signals to the device, it is possible to obtain specific balanced ternary data streams (such as “6C5F” and “5357”). Subsequently, the balanced ternary information is converted into binary information through programming, then transformed into the corresponding numbers and letters based on ASCII code, and finally converted into Chinese characters via Unicode, as shown in Figure 5b. Thus, the WSe_2_ SFG‐PD device, due to its high‐speed response and excellent safety, shows great promise in the field of optical‐electrical hybrid communication.

## 3 Conclusion

By leveraging the unique properties of 2D materials, we designed and fabricated the WSe_2_ SFG‐PD device by stacking 2D layered structures of WSe_2_, h‐BN, and graphene. By manipulating the voltage pulses of the back gate, charge carriers can be stored and erased in the graphene floating gate. Using this approach, we successfully achieved a programmable non‐volatile self‐driven photodetector with ultra‐fast response/recovery speeds (8.77 µs /2.02 µs and 5.92 µs /7.80 µs), high responsivity (2.76 A/W and 1.63 A/W), high specific detectivity (7.86 × 10^11^ Jones and 4.42 × 10^11^ Jones), low noise equivalent power (4.76 × 10^−15^ W/Hz^1/2^ and 8.47 × 10^−15^ W/Hz^1/2^), external quantum efficiency (537% and 318%), and excellent retention performance (>2 × 10^3^ s). In addition, the device is capable of generating relatively symmetric, oppositely directed built‐in electric fields (p^+^‐p/n‐p junctions). Based on this, we achieved dual‐channel optoelectronic hybrid communication by generating balanced ternary electrical signals, enabling high speed, large capacity, low loss, and high security. This demonstrates that the WSe_2_ SFG‐PD device offers a fast, effective, and convenient new option for optoelectronic hybrid communication.

## 4 Experimental Section

### Device Fabrication

SiO_2_/Si (with 300 nm SiO_2_ grown on a p^+^‐doped Si substrate) was chosen as the substrate. In the initial step, the substrate was first cleaned ultrasonically in acetone, ethanol, and deionized water for 5 min, followed by nitrogen flow drying to remove surface contaminants, ensuring a smooth and even surface. Next, the 2D materials were transferred onto a designated transfer platform. All 2D material flakes (WSe_2_, h‐BN, and graphene) were mechanically exfoliated from bulk crystals using polydimethylsiloxane (PDMS). Using a dry transfer technique, in a typical process, graphene flakes were first exfoliated onto a silicon wafer coated with a 300 nm thick layer of thermally oxidized SiO_2_. Then, h‐BN flakes were transferred onto a transparent PDMS film using adhesive tape and aligned on the graphene flakes with the aid of an optical microscope. By applying gentle pressure, the h‐BN was separated from the PDMS film and transferred onto the graphene. Similarly, the WSe_2_ layer was aligned and transferred onto the h‐BN (with some regions of WSe_2_ above graphene and others without). Subsequently, the materials on the substrate and the device were annealed in a nitrogen atmosphere at 250 °C for 3 h to remove interfacial impurities and achieve vdWs contacts between the layers. Finally, using a UV maskless lithography system (Suzhou TuoTuo Technology Co., Ltd.), Se/Au (5 nm/50 nm) electrodes were fabricated via metallic thermal evaporation, and the device was annealed in a vacuum environment at 210 °C (4 × 10^−4 ^Pa) for 6 h to remove the sacrificial Se layer, resulting in the WSe_2_ SFG‐PD device.

### Characterization and Electrical Testing

Atomic force microscopy (AFM) using the Dimension Fast Scan AFM from BRUKER was employed to characterize the thickness of the 2D materials (WSe_2_, h‐BN, and graphene). Raman spectra were collected using Raman microscope system (the Renishaw InVia) under 532 nm laser excitation. The optical response characteristics of the WSe_2_ SFG photodetector at different wavelengths of the laser system were measured in ambient conditions, both in darkness and under illumination, using a Keithley 2634 B analyzer. An optical attenuator and power meter were used to control and record the light intensity.

## Conflict of Interest

The authors declare no conflict of interest.

## Supporting information



Supporting Information

## Data Availability

The data that support the findings of this study are available from the corresponding author upon reasonable request.

## References

[1] a) D. Bertozzi , G. Dimitrakopoulos , J. Flich , S. Sonntag , Design Automation Embedded Syst. 2015, 19, 59;

[2] a) K. S. Novoselov , V. I. Fal'ko , L. Colombo , P. R. Gellert , M. G. Schwab , K. Kim , Nature 2012, 490, 192; 23060189 10.1038/nature11458

[3] a) F. H. L. Koppens , T. Mueller , P. Avouris , A. C. Ferrari , M. S. Vitiello , M. Polini , Nat. Nanotechnol. 2014, 9, 780; 25286273 10.1038/nnano.2014.215

[4] a) Q. H. Wang , K. Kalantar‐Zadeh , A. Kis , J. N. Coleman , M. S. Strano , Nat. Nanotechnol. 2012, 7, 699; 23132225 10.1038/nnano.2012.193

[5] D. Li , M. Y. Chen , Z. Z. Sun , P. Yu , Z. Liu , P. M. Ajayan , Z. X. Zhang , Nat. Nanotechnol. 2017, 12, 901. 28604709 10.1038/nnano.2017.104

[6] F. Liu , X. Lin , Y. Yan , X. Gan , Y. Cheng , X. Luo , Nano Lett. 2023, 23, 11645. 38088857 10.1021/acs.nanolett.3c03500

[7] D. Li , M. Chen , Q. Zong , Z. Zhang , Nano Lett. 2017, 17, 6353. 28956929 10.1021/acs.nanolett.7b03140

[8] a) F. Gong , W. Deng , Y. Wu , F. Liu , Y. Guo , Z. Che , J. Li , J. Li , Y. Chai , Y. Zhang , Nano Res. 2024, 17, 3113;

[9] B. W. H. Baugher , H. O. H. Churchill , Y. F. Yang , P. Jarillo‐Herrero , Nat. Nanotechnol. 2014, 9, 262. 24608231 10.1038/nnano.2014.25

[10] M. Buscema , D. J. Groenendijk , G. A. Steele , H. S. J. van der Zant , A. Castellanos‐Gomez , Nat. Commun. 2014, 5, 4651. 25164986 10.1038/ncomms5651

[11] Q. R. Deng , T. Zhao , J. L. Zhang , W. B. Yue , L. Li , S. S. Li , L. Y. Zhu , Y. M. Sun , Y. Pan , T. Zheng , X. T. Liu , Y. Yan , N. J. Huo , ACS Nano 2024, 18, 23702. 39147598 10.1021/acsnano.4c08345

[12] C. Liu , X. Yan , X. Song , S. Ding , D. W. Zhang , P. Zhou , Nat. Nanotechnol. 2018, 13, 404. 29632398 10.1038/s41565-018-0102-6

[13] M. S. Choi , G.‐H. Lee , Y.‐J. Yu , D.‐Y. Lee , S. Hwan Lee , P. Kim , J. Hone , W. J. Yoo , Nat. Commun. 2013, 4, 1624. 23535645 10.1038/ncomms2652

[14] A. J. Hong , E. B. Song , H. S. Yu , M. J. Allen , J. Kim , J. D. Fowler , J. K. Wassei , Y. Park , Y. Wang , J. Zou , R. B. Kaner , B. H. Weiller , K. L. Wang , ACS Nano 2011, 5, 7812. 21854056 10.1021/nn201809k

[15] W. Li , J. Y. Li , T. H. Mu , J. Y. Li , P. C. Sun , M. J. Dai , Y. H. Chen , R. J. Yang , Z. Chen , Y. C. Wang , Y. P. Wu , S. X. Wang , Small 2024, 20, 13.

[16] E. X. Wu , Y. Xie , S. J. Wang , D. H. Zhang , X. D. Hu , J. Liu , Nanoscale 2020, 12, 18800. 32970061 10.1039/d0nr03965a

[17] a) E. X. Wu , Y. Xie , J. Zhang , H. Zhang , X. D. Hu , J. Liu , C. W. Zhou , D. H. Zhang , Sci. Adv. 2019, 5, 9; 10.1126/sciadv.aav3430 PMC6499594 31058220

[18] a) J. J. Zha , S. H. Shi , A. Chaturvedi , H. X. Huang , P. Yang , Y. Yao , S. Y. Li , Y. P. Xia , Z. M. Zhang , W. Wang , H. D. Wang , S. C. Wang , Z. Yuan , Z. B. Yang , Q. Y. He , H. L. Tai , E. H. T. Teo , H. Y. Yu , J. C. Ho , Z. R. Wang , H. Zhang , C. L. Tan , Adv. Mater. 2023, 35, 12; 10.1002/adma.202211598 36857506

[19] Y.‐N. Xu , W. Y. Ching , Phys. Rev. B 1991, 44, 7787. 10.1103/physrevb.44.7787 9998708

[20] a) M. Lenzlinger , E. H. Snow , IEEE Trans. Electron Devices 1968, 15, 686;

[21] a) W. Liu , J. H. Lv , L. Peng , H. W. Guo , C. Liu , Y. L. Liu , W. Li , L. F. Li , L. X. Liu , P. Q. Wang , S. C. Bodepudi , K. Shehzad , G. H. Hu , K. H. Liu , Z. P. Sun , T. Hasan , Y. Xu , X. M. Wang , C. Gao , Y. Bin , X. F. Duan , Nat. Electron. 2022, 5, 281;

[22] a) J. Huang , K. Shu , N. Bu , Y. Yan , T. Zheng , M. Yang , Z. Zheng , N. Huo , J. Li , W. Gao , Sci. China Mater. 2023, 66, 4711;

[23] a) T. Zheng , M. Yang , Y. Pan , Z. Zheng , Y. Sun , L. Li , N. Huo , D. Luo , W. Gao , J. Li , ACS Appl. Mater. Interfaces 2023, 15, 29363; 37294943 10.1021/acsami.3c04147

[24] Z. Wang , Q. Li , Y. Chen , B. Cui , Y. Li , F. Besenbacher , M. Dong , NPG Asia Mater. 2018, 10, 703.

