# Supplementary material for: 2D Programmable Photodetectors Based on WSe2/h‐BN/Graphene Heterojunctions
Source: Adv Sci (Weinh). 2025 Apr 4;12(22):2417300. doi: 10.1002/advs.202417300 (PMC12165107; doi:10.1002/advs.202417300)
Supplement: Supplementary file 1 — Supporting Information [file ADVS-12-2417300-s001.docx]

Supporting Information

**Two Dimensional Programmable Photodetectors Based on WSe_2_/h-BN/Graphene Heterojunctions**

*Zhihao Wang, Jialing Jian, Zhengjin Weng, Qianqian Wu, Jian Li, Xingyu Zhou,* *Wei Kong, Xiang Xu, Liangliang Lin, Xiaofeng Gu, Peng Xiao*, Haiyan Nan*, and Shaoqing Xiao**

**KPFM characterization of WSe_2_/h-BN/graphene**

Kelvin Probe Force Microscopy (KPFM) maps the surface potential with nanometer-scale spatial resolution, allowing the study of the electrical properties of material interfaces. Therefore, KPFM is used to determine the surface potential of WSe_2_ under UV irradiation.


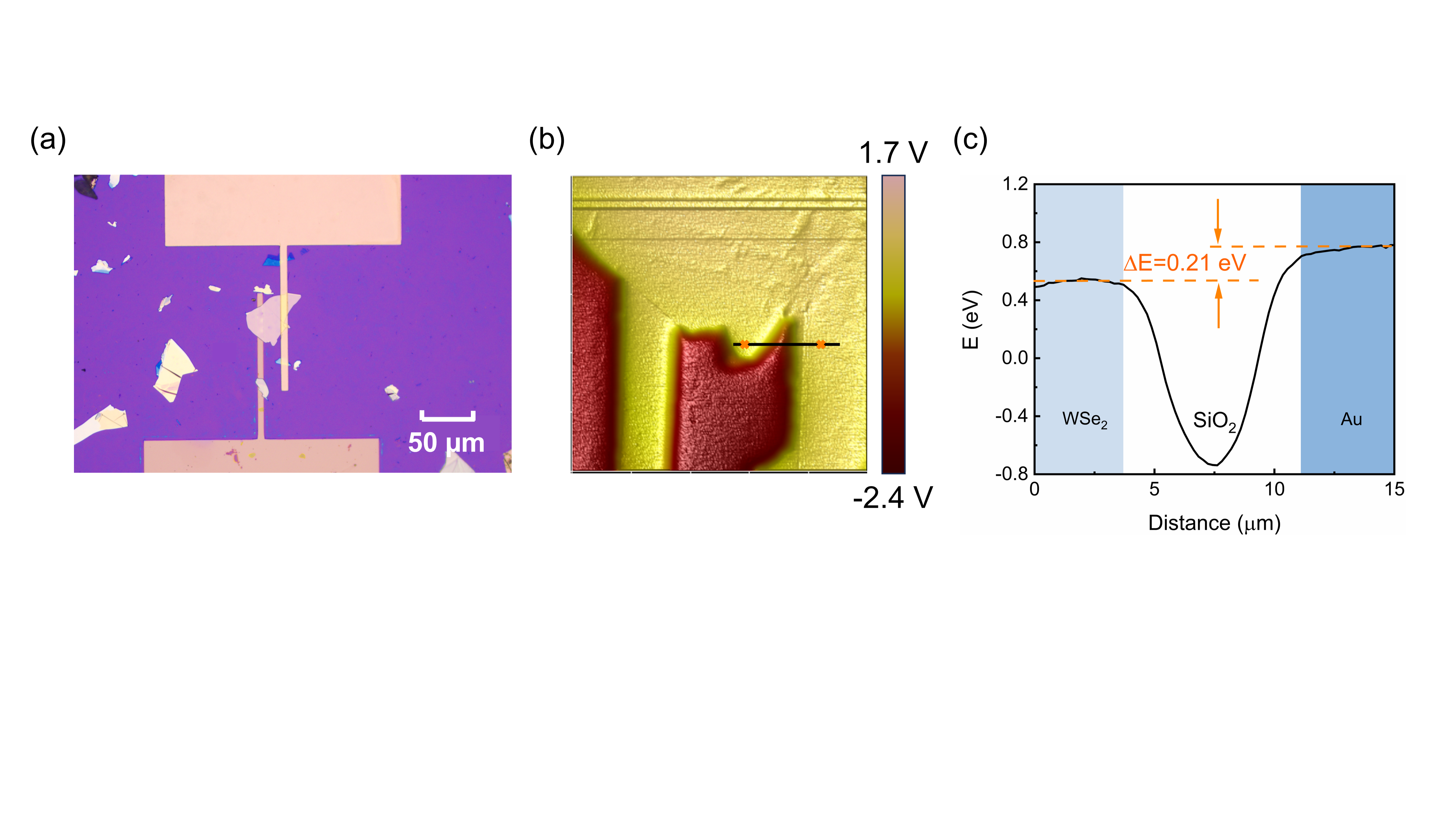


**Figure S1.** (a) The optical image of the Au-WSe_2_-Au device. (b) The 3D device image of the device measured under KPFM (with the extraction range indicated by black lines). (c) The difference between W_WSe2_ and W_Au_, with a value of 0.21 eV. W_WSe2_ = W_Au_ – W_CPD_ = 5.1 eV – 0.21 eV = 4.89 eV.

**Schematic diagram of the process flow of WSe_2_/h-BN/graphene heterojunction**


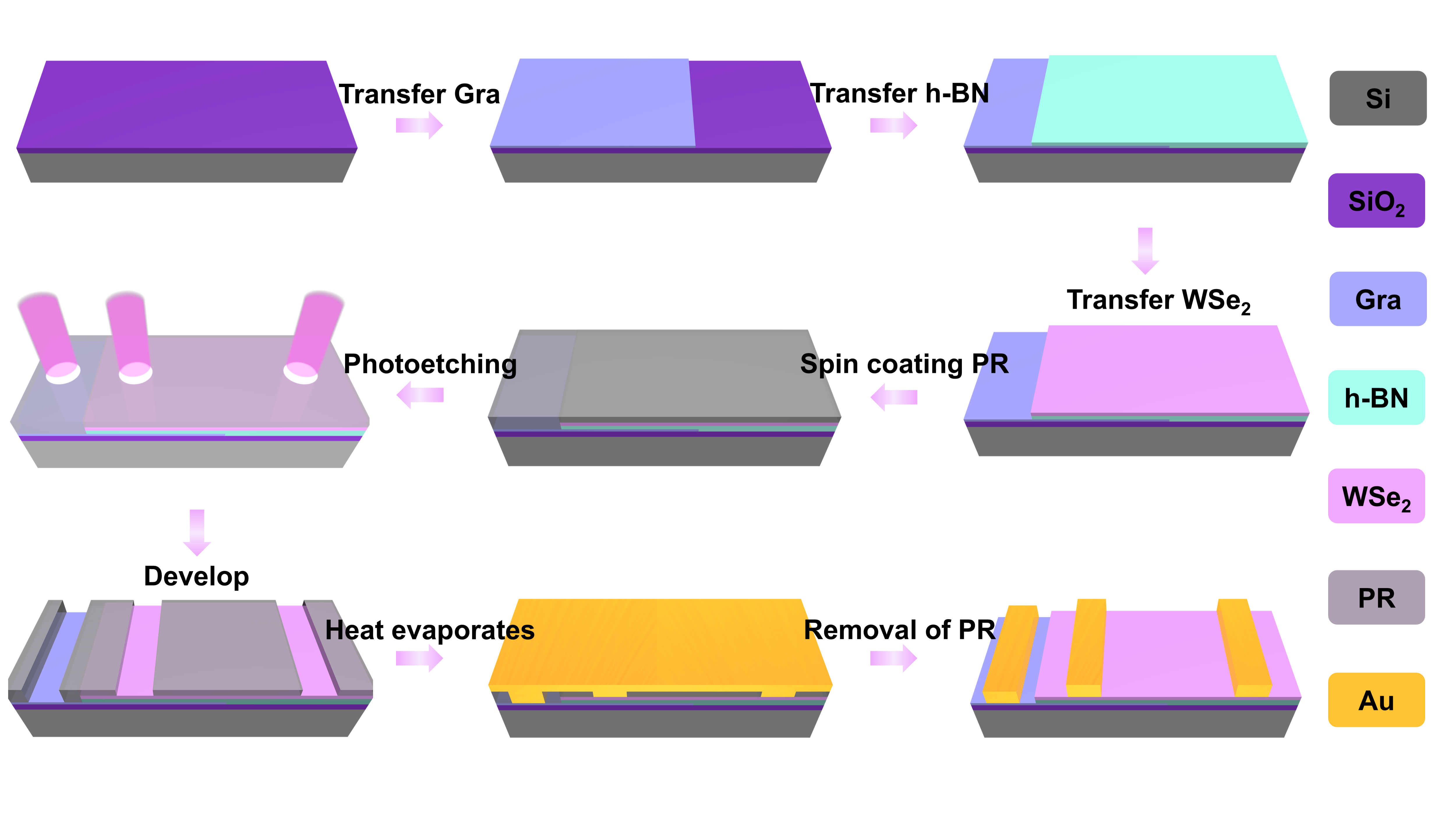


**Figure S2.** Schematic diagram of the manufacturing process for the WSe_2_ SFG-PD structure.

The manufacturing process of the WSe_2_ SFG-PD structure illustrated in Figure S2. Here, Gra represents graphene and PR represents photoresist.

**AFM characterization of WSe_2_/h-BN/graphene**


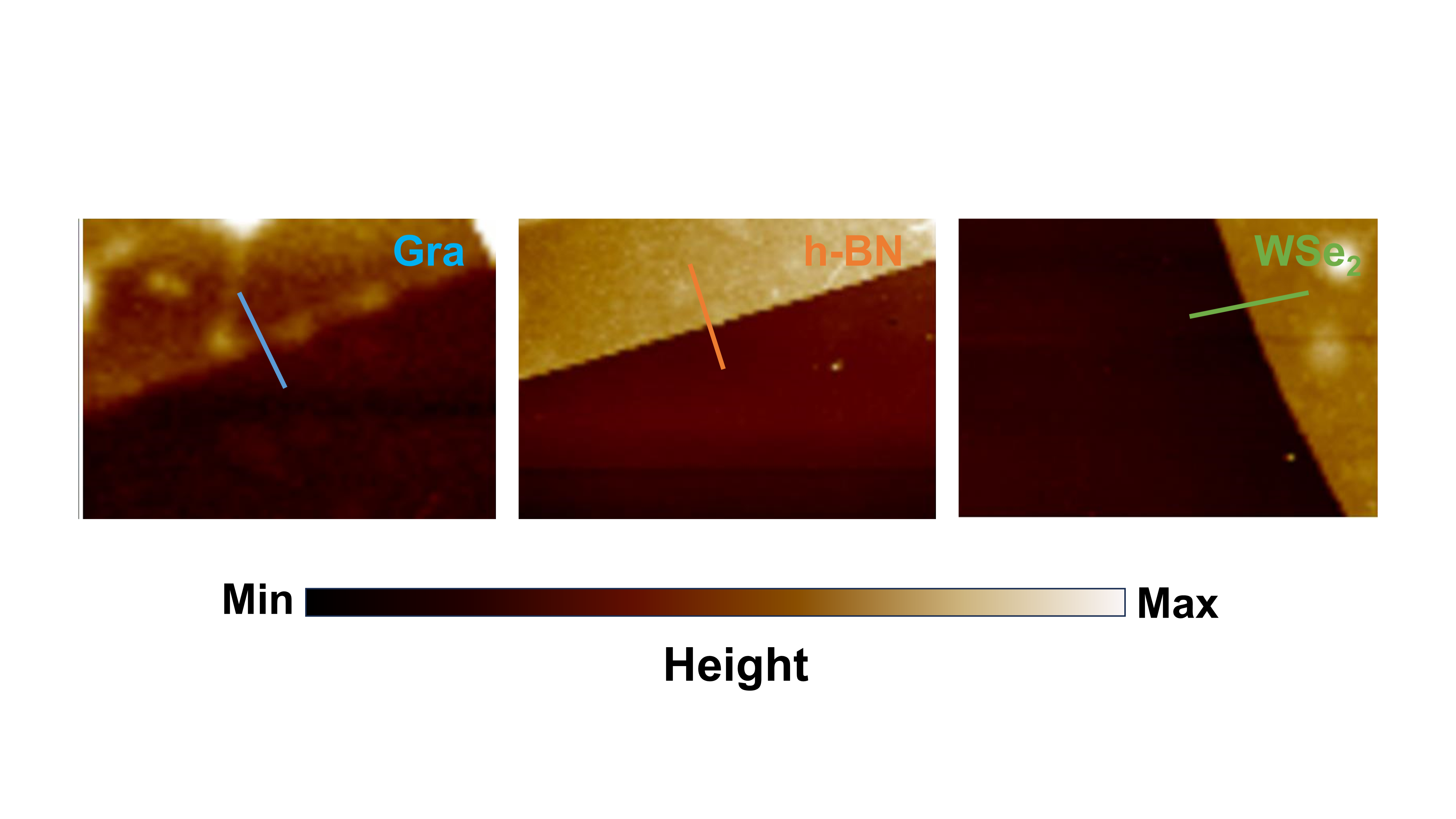


**Figure S3.** AFM image of the WSe_2_ SFG-PD device. The blue, orange, and green lines correspond to the extracted heights of Gra, h-BN, and WSe_2_ flakes, respectively.


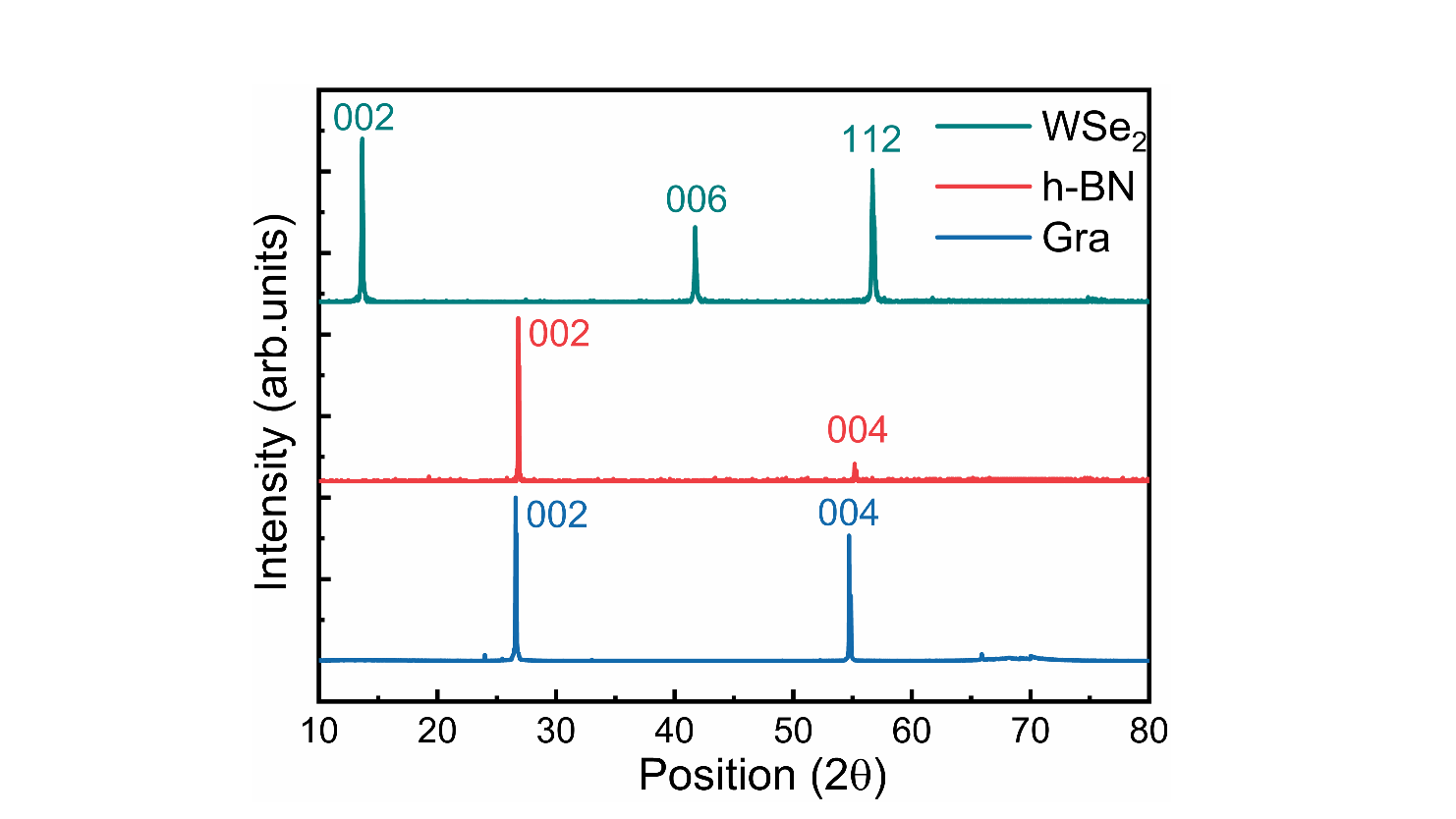


**Figure S4.** The X-ray diffraction (XRD) spectra of WSe_2_, h-BN, and Gra are shown, with the blue, red, and green curves corresponding to the XRD patterns of graphene, h-BN, and WSe_2_, respectively.

The WSe_2_ flakes exhibit three distinct diffraction peaks at 13.6°, 41.7°, and 56.6°, corresponding to the (002), (006), and (112) planes, respectively. The characteristic diffraction peaks of h-BN are located at 26.8° and 55.1°, corresponding to the (002) and (004) planes, respectively. Additionally, typical diffraction peaks of multi-layer graphene are observed at 26.5° (002) and 54.7° (004). These results indicate that the materials exhibit an ordered layered structure, good interlayer stacking, few defects, and high crystallinity, further confirming their high-quality characteristics.

**The electronic performance**


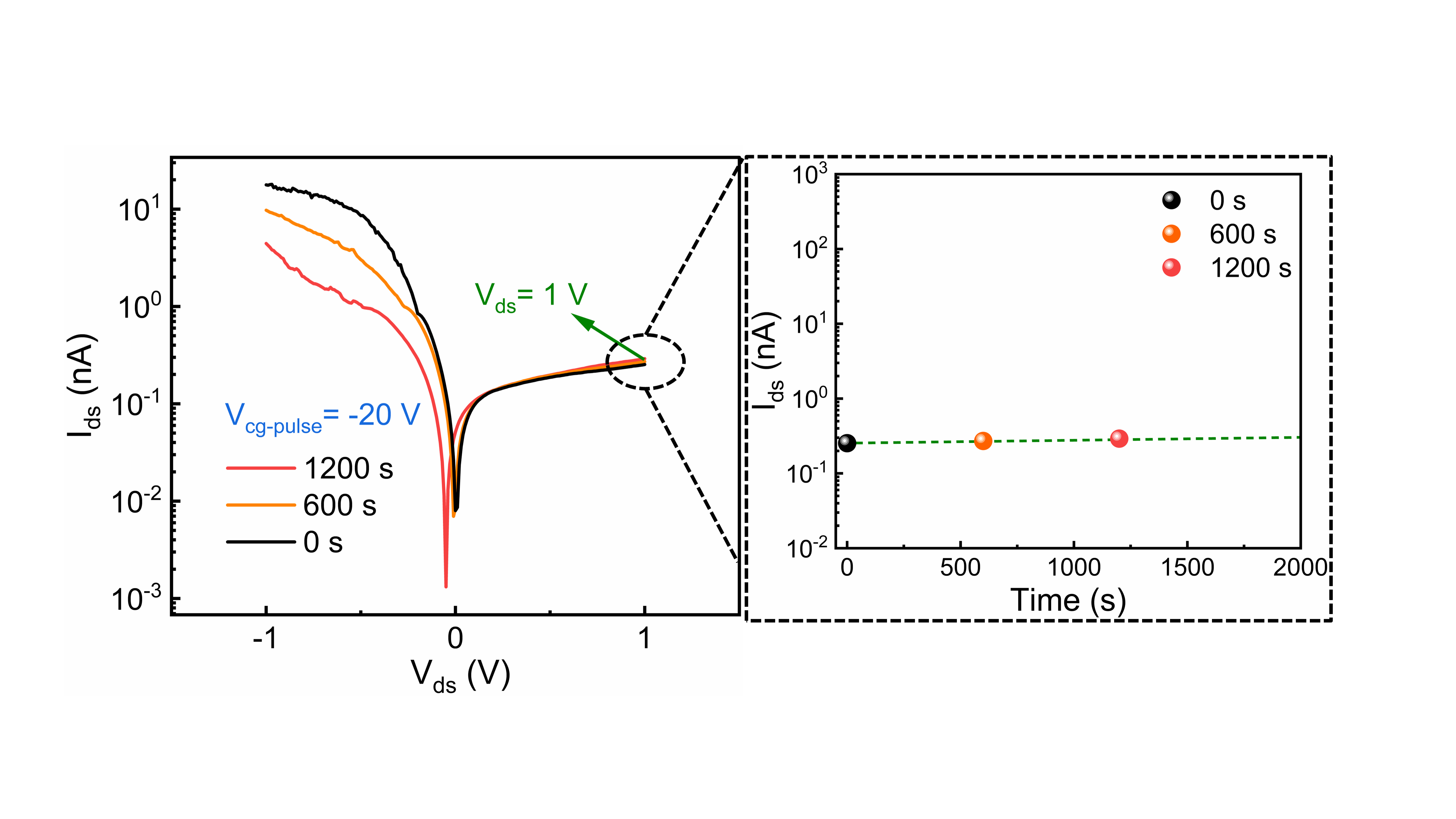


**Figure S5.** Left image: Time-dependent semi-logarithmic I_DS_-V_DS_ curve of WSe_2_ under a -20 V gate voltage pulse applied to Si. Right image: Fitting curve of I_DS_ as a function of time for WSe_2_ under a -20 V gate voltage pulse with V_DS_ = +1 V applied on Si.

It can be clearly seen from the right graph of Figure S5 that at V_cg-pulse_ = -20 V, I_DS(VDS = 1 V)_ remains stable around 0.15 nA for up to 2000 seconds. This indicates that the SFG can store and maintain tunneling holes for a long period, demonstrating the excellent stability of the device.


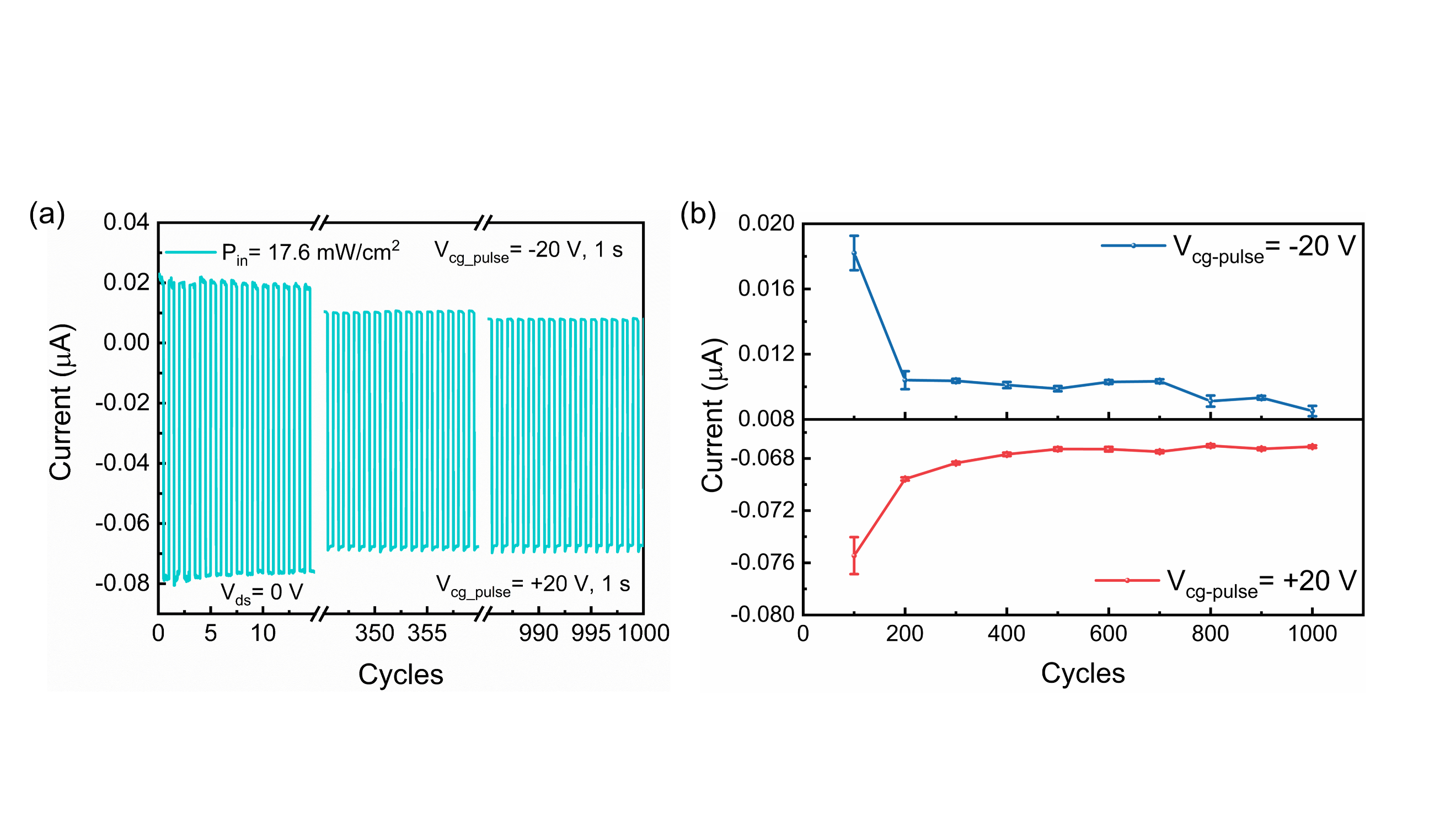


**Figure S6.** (a) Presents the repeatability test curve of the WSe_2_ SFG-PD device, and (b) shows the corresponding error bars.

To further assess the device’s stability, we tested the performance of the WSe_2_ SFG-PD device under 1000 cycles of periodic gate voltage pulses, as shown in Figure S6. With an incident light power of 1.76 mW/cm² and a ±20 V gate voltage, the device continues to demonstrate stable and reversible photoresponse after 1000 gate voltage pulse cycles (Figure S6a), with the error remaining within 1 nA (Figure S6b).


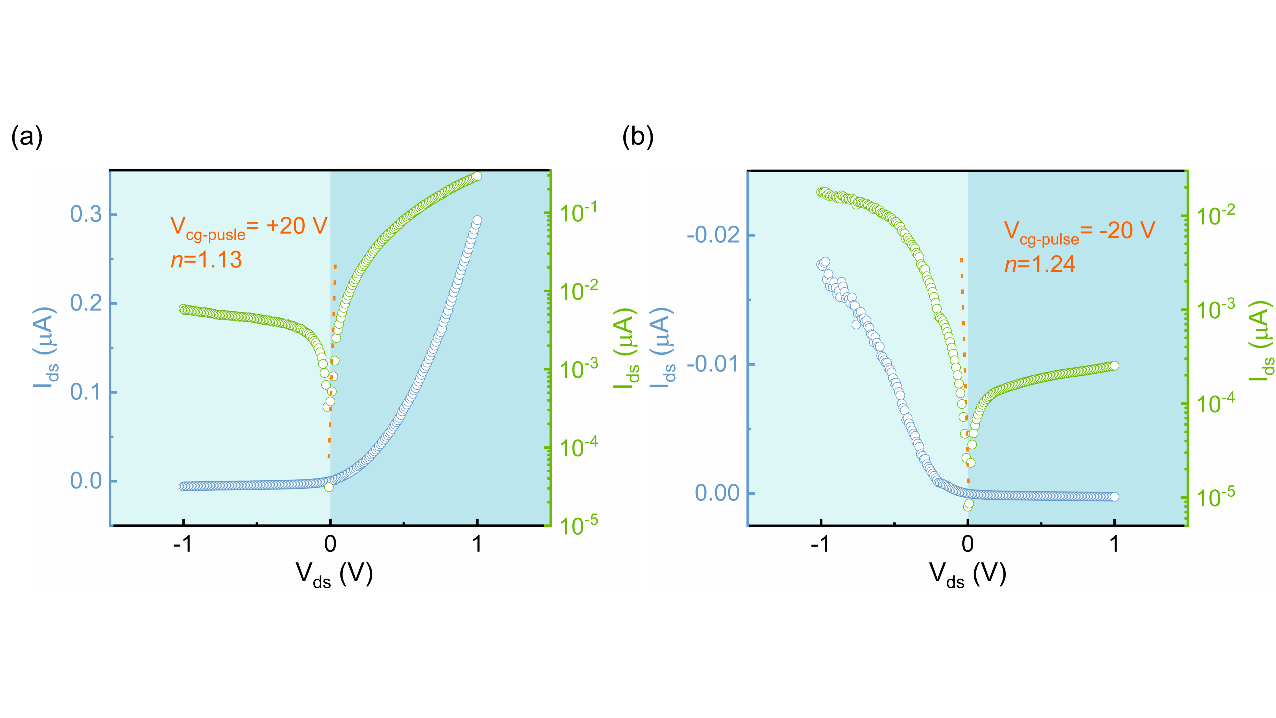


**Figure S7.** (a-b) Displaying the linear (left axis) and semi-logarithmic (right axis) curves of I_DS_-V_DS_ for WSe_2_ under ±20 V gate pulse applied on Si, varying with time.

As shown in Figure S7, the ideal factors n for the WSe_2_ SFG-PD devices are 1.13 and 1.24 at V_cg-pulse_ = ±20 V, respectively.


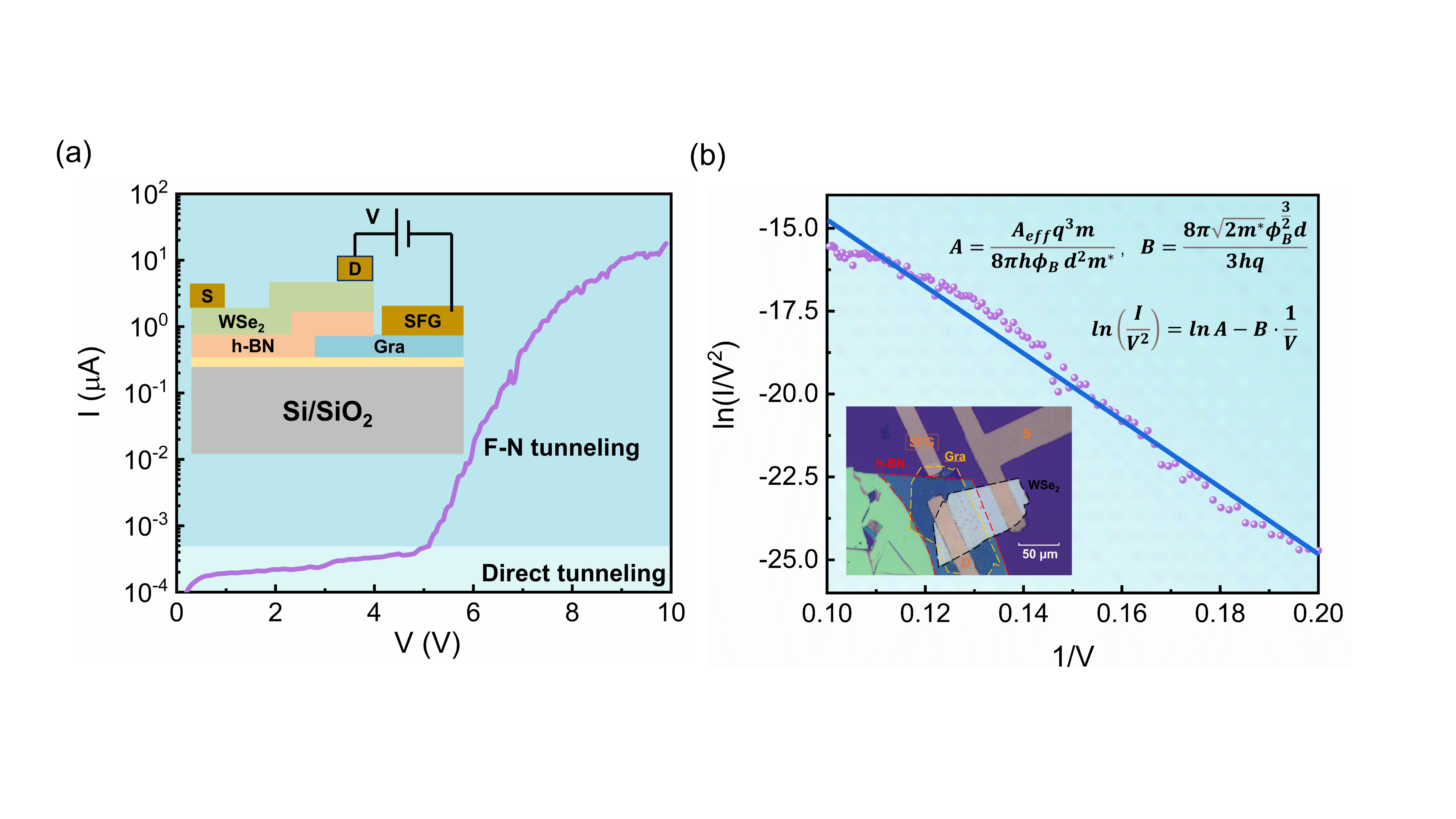


**Figure S8.** (a) The relationship between the measured tunneling current and the voltage V. The inset depicts a schematic of the test structure, where V is the voltage applied to electrode D (drain) and the SFG. (b) The Fowler−Nordheim (F−N) plot at high bias. The inset shows an optical image of the device.

**The optical performance**


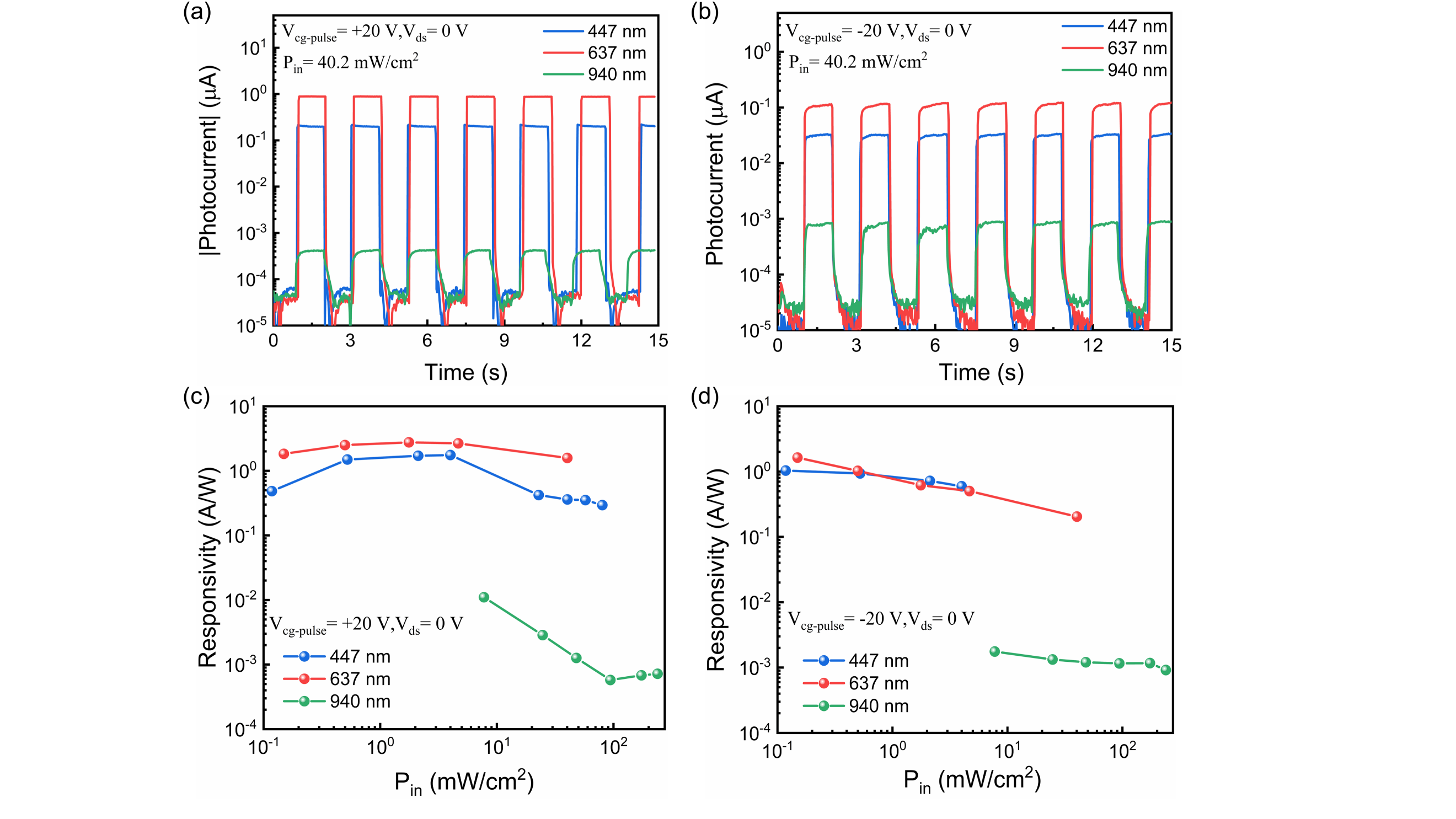


**Figure S9.** (a-b) Show the time-dependent photoresponse under illumination with different wavelengths for V_cg-pulse_ = +20 V and V_cg-pulse_ = -20 V, respectively. As illustrated in the figures, the photocurrent of the WSe_2_ SFG-PD device is higher under 637 nm illumination compared to the other two wavelengths. (c-d) Present the wavelength-dependent responsivity for V_cg-pulse_ = +20 V and V_cg-pulse_ = -20 V, respectively. The results indicate that the WSe_2_ SFG-PD device achieves the highest responsivity under 637 nm illumination.





**Figure S10.** Semi-logarithmic plot of the output curve under 637 nm illumination, with a -20 V voltage pulse applied to the Si substrate with 0 V bias at different input optical power levels (P_in_).


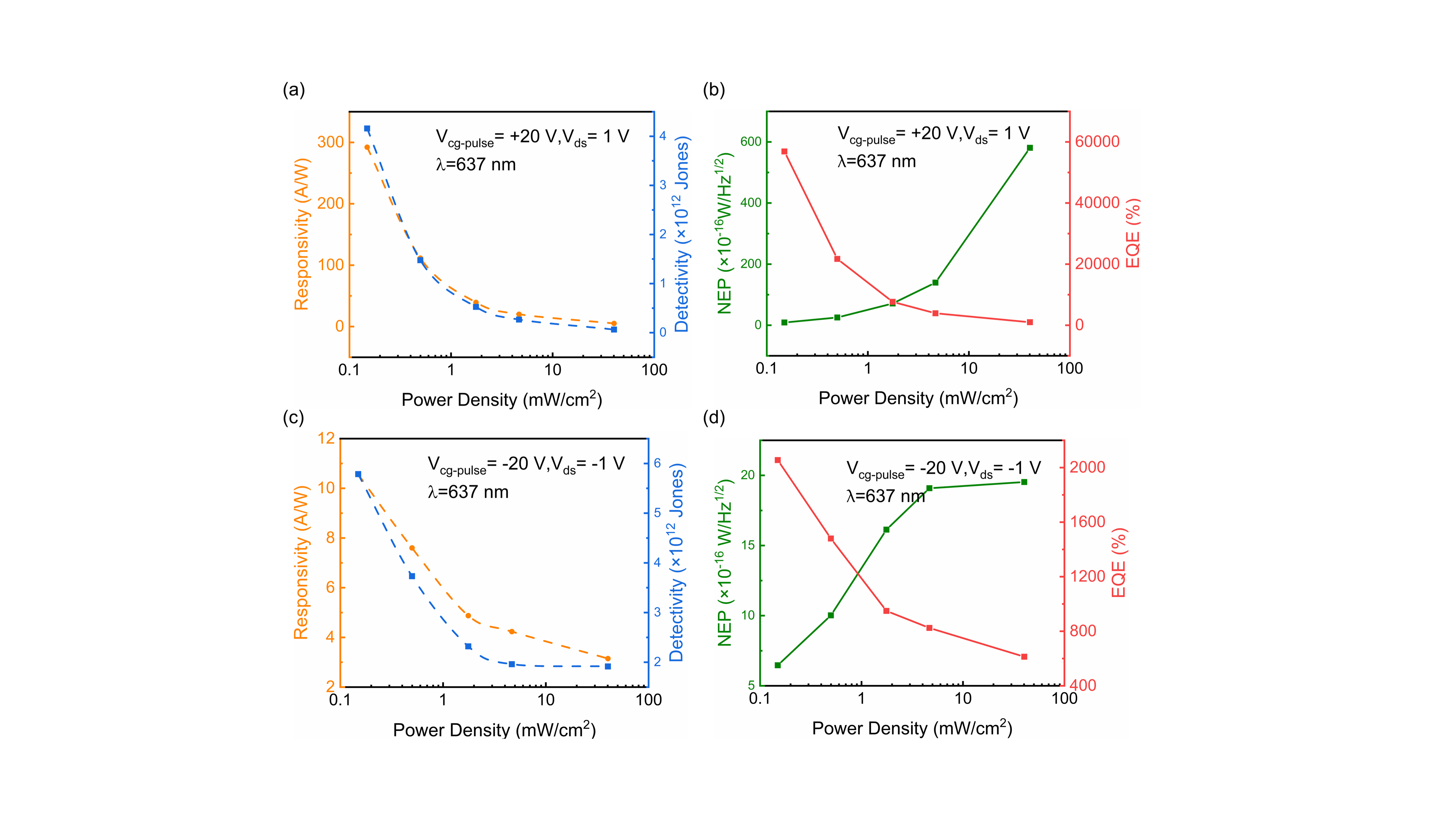


**Figure S11.** (a, b) The optoelectronic performance of the device at V_cg-pulse_ = +20 V, 1 V bias, and 637 nm, corresponding to the responsivity R, detectivity D*, noise equivalent power NEP, and external quantum efficiency EQE, respectively. (c, d) The optoelectronic performance of the device at V_cg-pulse_ = -20 V, -1 V bias, and 637 nm, corresponding to the responsivity R, detectivity D*, noise equivalent power NEP, and external quantum efficiency EQE, respectively.

We quantitatively evaluated the optoelectronic detection performance of the device using key performance indicators such as Responsivity (R), Specific Detectivity (D*), External Quantum Efficiency (EQE), and Noise Equivalent Power (NEP). The formulas used are as follows^[1]^:

$R_{\lambda}=\frac{I_{light}-I_{dark}}{P_{in}\times S}$ (1)

$D^{*}=R\sqrt{S/2qI_{dark}}$ (2)

$EQE=\frac{{(I}_{light}-I_{dark)/q}}{P_{in}/(hv)}=R\frac{\mathrm{hc}}{q\lambda}$ (3)

$NEP=\frac{S^{1/2}}{D^{*}}$ (4)

In this context, *I_light_*, *I_dark_*, *P_in_*, and *S* represent the photocurrent, dark current, incident laser power density (based on the effective illuminated area of the device), and the effective light area of the device, respectively. *υ* is the frequency of the incident light, *λ* is the wavelength of the incident light, q is the elementary charge, c is the speed of light in vacuum, and h is Planck's constant.

When V_cg-pulse_ = +20 V and V_ds_ = 1 V, the maximum responsivity is 292.2 A/W, the maximum detectivity is 4.16 × 10^12^ Jones, the minimum noise equivalent power is 8.99 × 10^-16^ W/Hz^1/2^, and the external quantum efficiency reaches up to 56900%. When V_cg-pulse_ = -20 V and V_ds_ = -1 V, the maximum responsivity is 10.56 A/W, the maximum detectivity is 5.79 × 10^12^ Jones, the minimum noise equivalent power is 6.47 × 10^-16^ W/Hz^1/2^, and the external quantum efficiency reaches up to 2060%.

**Comparison of the contact effects between WSe_2_ and Au electrodes via direct and indirect thermal evaporation**


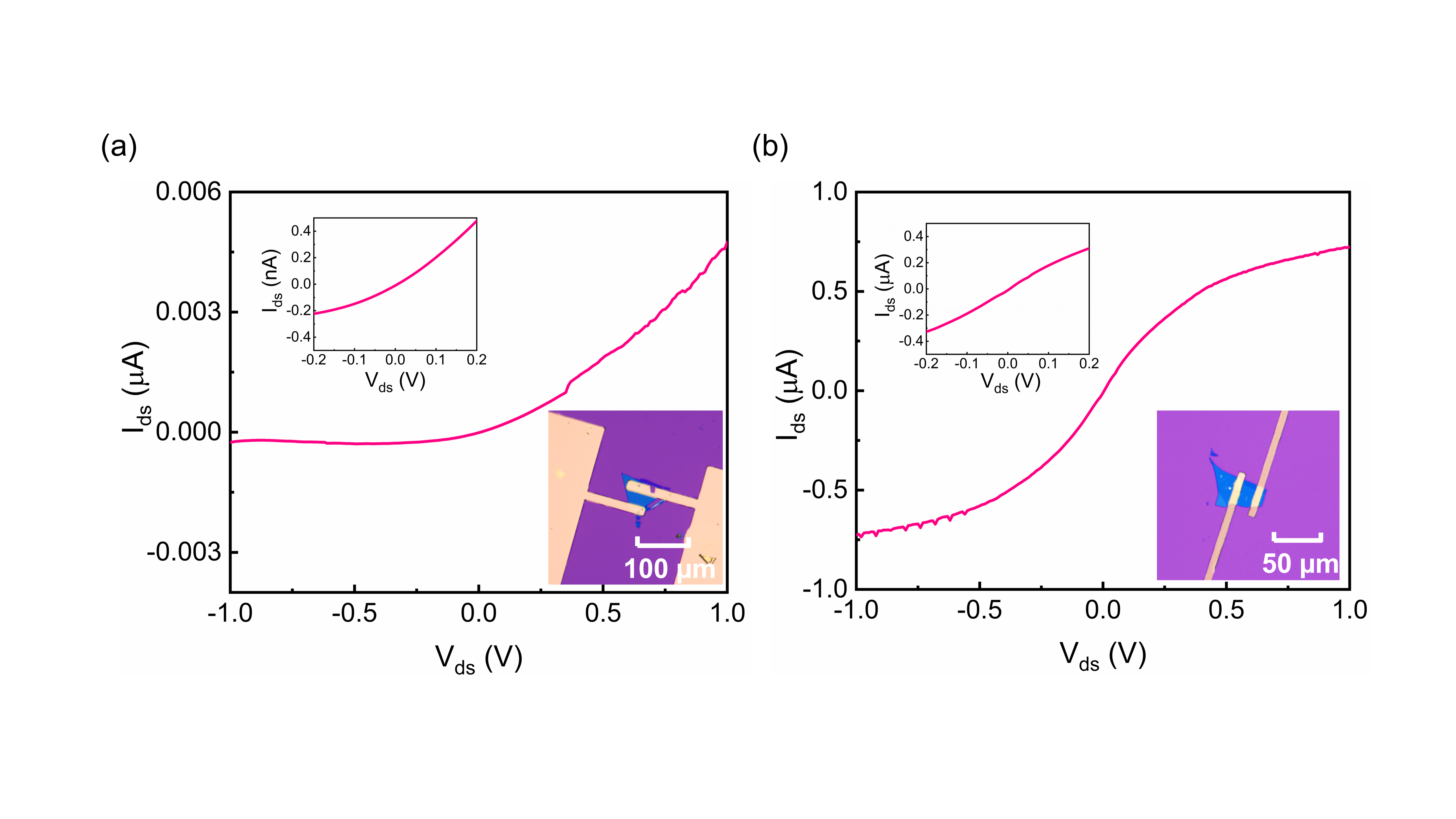


**Figure S12.** Output curves of the Au-WSe_2_-Au device are presented. (a) The Au electrode (50 nm) thermally deposited directly onto the WSe_2_ target. (b) The process where a sacrificial layer of Se (5 nm) is first thermally evaporated on the WSe_2_ target, followed by the thermal deposition of the Au electrode (50 nm). Finally, the sample is annealed for 6 hours in a vacuum environment (4 × 10^-4^ Pa) to remove the sacrificial Se layer. The inset shows an enlarged view of the original I_ds_ within the range of V_ds_ = ±0.2 V.

The asymmetric contact at both ends of the electrode and the small current magnitude are evident in Figure S12 (a), possibly due to some Au atoms entering WSe_2_  and introducing defects that result in varying degrees of Fermi pinning.^[2]^ As shown in Figure S12 (b), the symmetric contact at both ends of the electrode and the increased current magnitude are evident, as the sacrificial Se layer effectively prevented Au atoms from entering WSe_2_ and introducing defects, thereby forming a vdWs metal-semiconductor contact.^[3]^

**The comparison of rectification effects in semi-floating gate photovoltaic devices fabricated from WSe_2_ materials of different thicknesses**


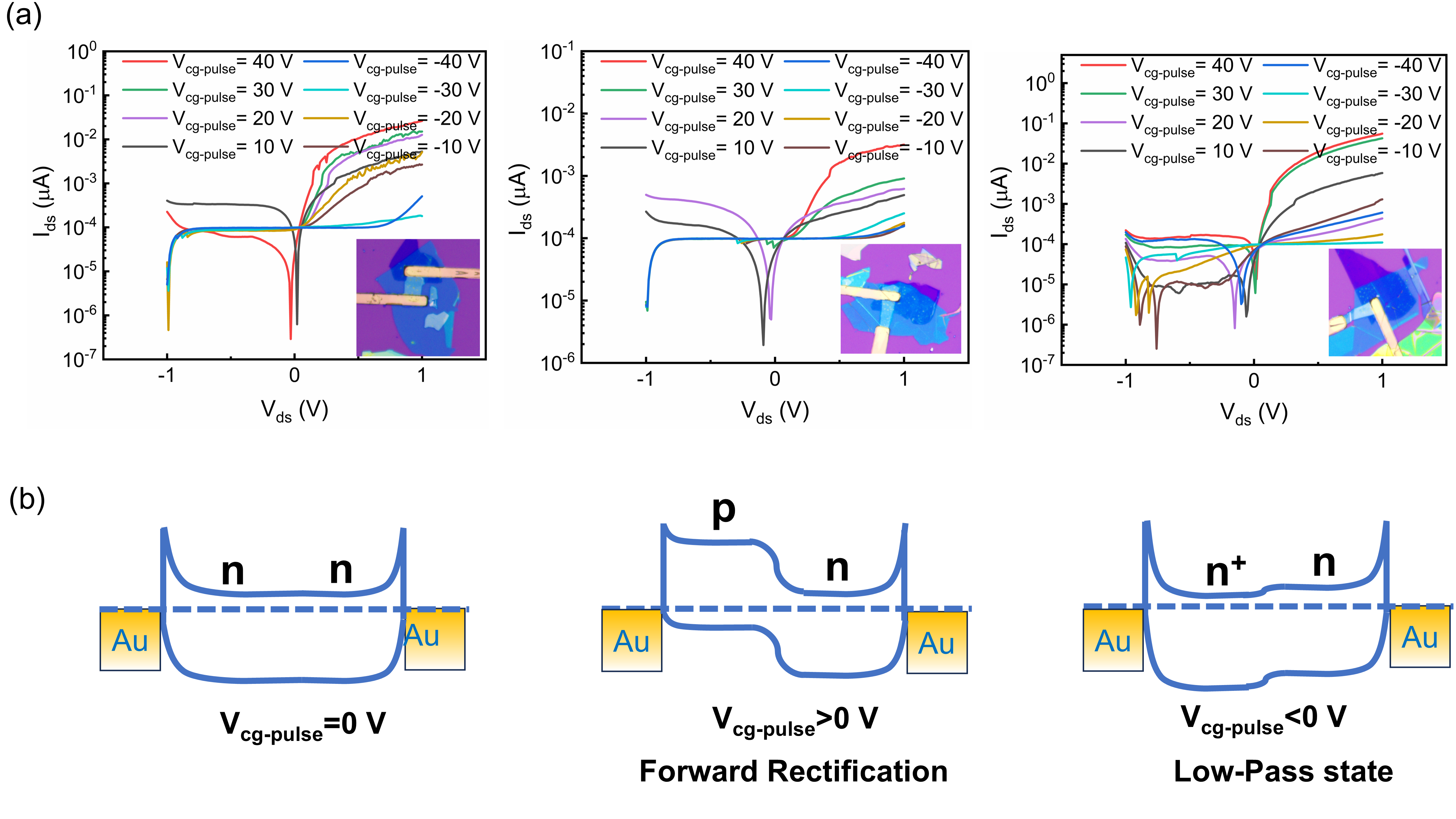


**Figure S13.** (a) The semi-logarithmic curves of I_DS_-V_DS_ measured on the WSe_2_ SFG-PD device fabricated with thin WSe_2_ (below 10 nm) under different gate voltage pulses applied to Si. The insets are optical images corresponding to the device. (b) A schematic diagram of the energy bands of WSe_2_ under different gate voltage pulses.

Figure S13 (a) clearly shows that as V_cg-pulse_ > 0 and the amplitude increases, the device exhibits forward rectification, with the rectification ratio continually increasing, reaching a maximum ratio of approximately 10^3^. Conversely, as V_cg-pulse_ < 0 and the amplitude increases, the rectification ratio of the device decreases, approaching a low-pass state. As shown in Figure S13 (b), the left panel shows the energy band diagram of WSe_2_ at V_cg-pulse_ = 0 V. The middle panel illustrates the energy band diagram of WSe_2_ at V_cg-pulse_ > 0 V, indicating that WSe_2_ can conduct unidirectionally to form forward rectification. The right panel displays the energy band diagram of WSe_2_ at V_cg-pulse_ < 0 V. Due to the presence of large Schottky barriers at both ends of the source and drain of WSe_2_, the leakage current is low, exhibiting a low-pass state.


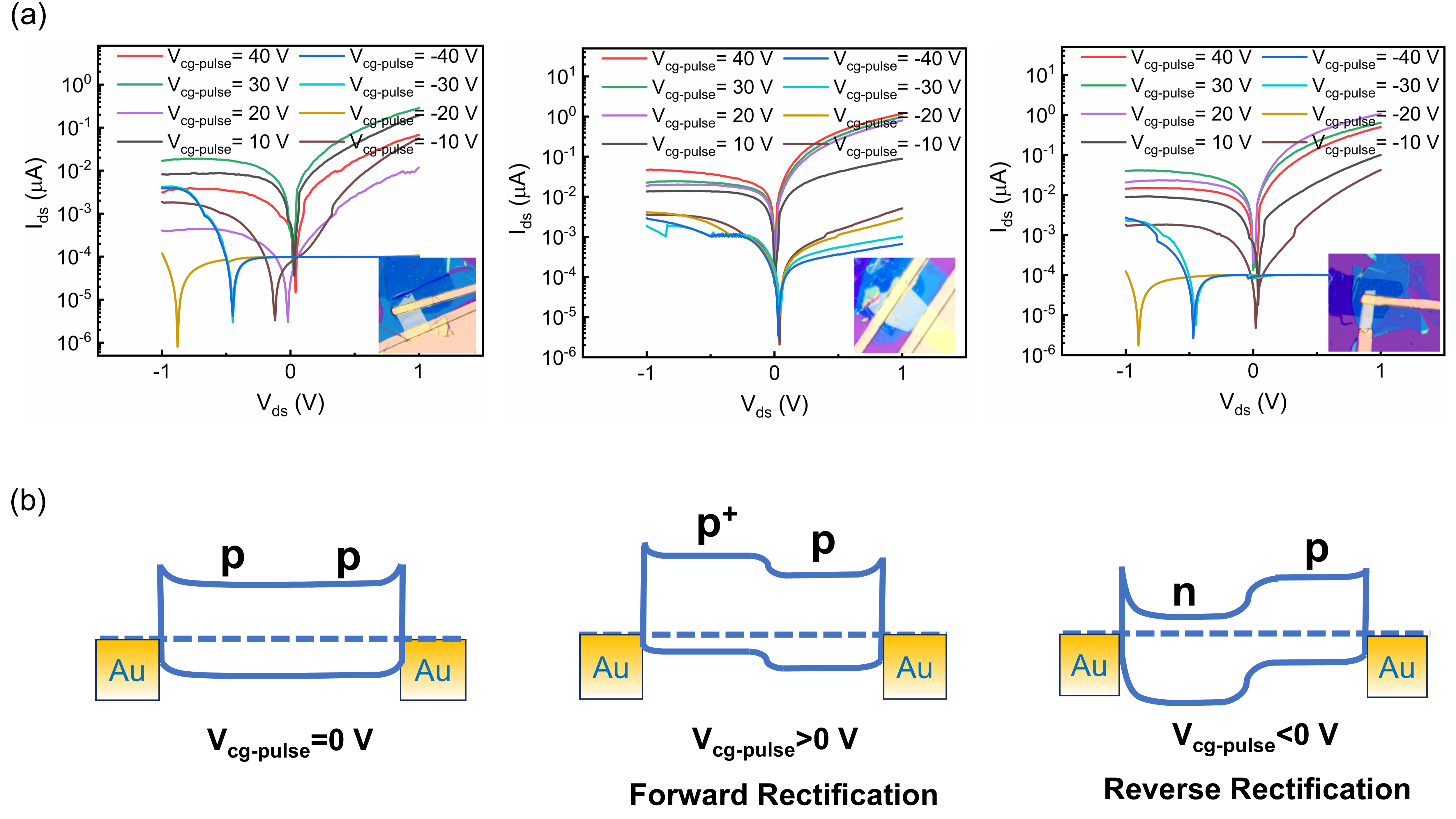


**Figure S14.** (a) The semi-logarithmic curves of I_DS_-V_DS_ measured on the WSe_2_ SFG-PD device fabricated with moderate thickness WSe_2_ (10 nm-30 nm) under different gate voltage pulses applied to Si. The insets are optical images corresponding to the device. (b) A schematic diagram of the energy bands of WSe_2_ under different gate voltage pulses.

Figure S14 (a) clearly shows that as V_cg-pulse_ > 0 and the amplitude increases, the device exhibits forward rectification, with the rectification ratio continually increasing, reaching a maximum ratio of approximately 10^2^. Conversely, as V_cg-pulse_ < 0 and the amplitude increases, the device exhibits reverse rectification, with the rectification ratio continually reducing, reaching a minimum ratio of approximately 10^-2^.As shown in Figure S14 (b), the left panel shows the energy band diagram of WSe_2_ at V_cg-pulse_ = 0 V. The middle panel illustrates the energy band diagram of WSe_2_ at V_cg-pulse_ > 0 V, indicating that WSe_2_ can conduct unidirectionally to form forward rectification. The right panel displays the energy band diagram of WSe_2_ at V_cg-pulse_ < 0 V, indicating that WSe_2_ can conduct unidirectionally to form reverse rectification.


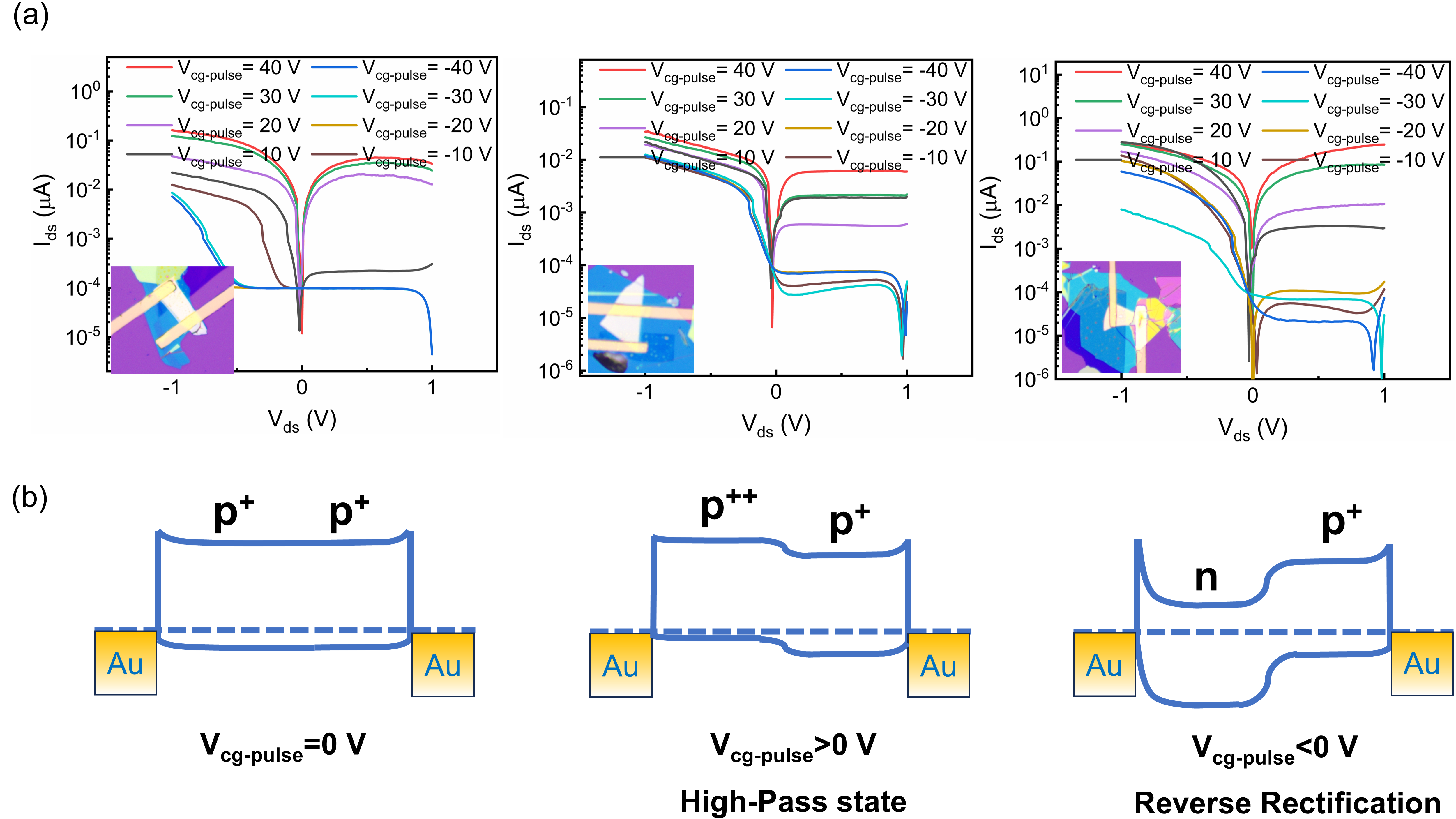


**Figure S15.** (a) The semi-logarithmic curves of I_DS_-V_DS_ measured on the WSe_2_ SFG-PD device fabricated with thick WSe_2_ (over 30 nm) under different gate voltage pulses applied to Si. The insets are optical images corresponding to the device. (b) A schematic diagram of the energy bands of WSe_2_ under different gate voltage pulses.

Figure S15 (a) clearly shows that as V_cg-pulse_ > 0 and the amplitude increases, the rectification effect of the device gradually diminishes, resulting in a high-pass current state. Conversely, as V_cg-pulse_ < 0 and the amplitude increases, the device exhibits reverse rectification, with the rectification ratio gradually reducing, reaching a minimum ratio of approximately 10^-3^. As shown in Figure S15 (b), the left panel shows the energy band diagram of WSe_2_ at V_cg-pulse_ = 0 V. The middle panel illustrates the energy band diagram of WSe_2_ at V_cg-pulse_ > 0 V, indicating that due to the smaller Schottky barrier at both ends of the source and drain, the leakage current is larger, resulting in a high-pass state. The right panel displays the energy band diagram of WSe_2_ at V_cg-pulse_ < 0 V, indicating that WSe_2_ can conduct unidirectionally and form reverse rectification.

**References**

[1] Q. Wu, C. Wang, L. Li, X. Zhang, Y. Jiang, Z. Cai, L. Lin, Z. Ni, X. Gu, K. Ostrikov, H. Nan, S. Xiao, *Journal of Materials Science & Technology* **2024**, 193, 217.

[2] a) C. Kim, I. Moon, D. Lee, M. S. Choi, F. Ahmed, S. Nam, Y. Cho, H.-J. Shin, S. Park, W. J. Yoo, *ACS Nano* **2017**, 11, 1588; b) J. Jang, Y. Kim, S.-S. Chee, H. Kim, D. Whang, G.-H. Kim, S. J. Yun, *ACS Applied Materials & Interfaces* **2020**, 12, 5031; c) M. J. Mleczko, A. C. Yu, C. M. Smyth, V. Chen, Y. C. Shin, S. Chatterjee, Y.-C. Tsai, Y. Nishi, R. M. Wallace, E. Pop, *Nano Letters* **2019**, 19, 6352; d) W. Zhou, X. Zou, S. Najmaei, Z. Liu, Y. Shi, J. Kong, J. Lou, P. M. Ajayan, B. I. Yakobson, J.-C. Idrobo, *Nano Letters* **2013**, 13, 2615; e) S. Kc, R. C. Longo, R. Addou, R. M. Wallace, K. Cho, *Nanotechnology* **2014**, 25, 375703; f) J. Hong, Z. Hu, M. Probert, K. Li, D. Lv, X. Yang, L. Gu, N. Mao, Q. Feng, L. Xie, J. Zhang, D. Wu, Z. Zhang, C. Jin, W. Ji, X. Zhang, J. Yuan, Z. Zhang, *Nature Communications* **2015**, 6, 6293.

[3] G. Kwon, Y.-H. Choi, H. Lee, H.-S. Kim, J. Jeong, K. Jeong, M. Baik, H. Kwon, J. Ahn, E. Lee, M.-H. Cho, *Nature Electronics* **2022**, 5, 241.
